# Poor treatment outcomes and its determinants among tuberculosis patients in selected health facilities in East Wollega, Western Ethiopia

**DOI:** 10.1371/journal.pone.0206227

**Published:** 2018-10-26

**Authors:** Abrham Belachew Muluye, Selamu Kebamo, Tesfa Teklie, Getachew Alemkere

**Affiliations:** Department of Pharmacy, College of Health Sciences, Wollega University, Nekemte, Ethiopia; Indian Institute of Technology Delhi, INDIA

## Abstract

**Background:**

Although it is a preventable and treatable disease, tuberculosis remains a major medical and public health problem throughout the world. The control and elimination of tuberculosis is currently challenged by the development and spread of antituberculosis drug resistance. The resistance is often correlated to the absence of properly implemented control measures that lead to poor treatment outcomes. Therefore, the aim of the current study was to assess poor treatment outcomes and its determinants among tuberculosis patients in selected health facilities in East Wollega zone, Western Ethiopia.

**Method:**

A five-year retrospective cross-sectional study design was employed. Data were collected from patients’ medical record from January to March 2017. Data were entered and analyzed using SPSS version 20. Descriptive statistics were used to generate and summarize frequencies. Univariate and multivariate logistic regression analysis were used to associate the potential determinants of poor treatment outcomes.

**Results:**

From 995 patients with documented treatment outcomes, 58.9% were males with a mean age of 31.9±16.3 years and 58% lived in rural areas. Majorities of cases (95.7%) were newly treated ones. Nearly half of the cases had extrapulmonary tuberculosis and 6.8% were co-infected with HIV. Nearly three-quarter of patients had completed their treatment while 17.2%, 2.9%, 4.8%, 0.4% patients were cured, defaulted, died, and failed, respectively. The overall treatment success rate was 91.9%. Being treated in Anger Gute health center (adjusted odds ratio (AOR): 2.27; 95% confidence interval (CI): 1.18–4.38); male (AOR: 1.81; 95% CI: 1.06–3.10); lived in rural areas (AOR: 1.73; 95% CI: 1.02–2.91); previously treated (AOR: 2.72; 95% CI: 1.16–6.39) and unknown HIV status (AOR: 4.56; 95% CI: 1.98–10.50) were determinants of poor treatment outcomes.

**Conclusion:**

The current treatment success rate was exceeded the recommended target. However, special attention and strict follow up is required for tuberculosis patients with high risk of unsuccessful treatment outcomes including male, rural resident, previously treated and unknown in HIV status patients throughout their treatment periods.

## Introduction

Infectious diseases are major causes of morbidity, mortality and poverty throughout the developing countries. Among these diseases, tuberculosis (TB) is an ancient and re-emergent bacterial disease that affects both humans and animals. It is mainly caused by the bacillus *Mycobacterium tuberculosis*. The bacteria infects about one third of the global population. It typically affects the pulmonary system (pulmonary TB) and occasionally any other anatomic site (extrapulmonary TB) of the body. The disease is mainly transmitted by inhaling droplet aerosols from lungs with active lung disease [1–4].

In 2016, tuberculosis was the ninth leading cause of global deaths. It was a number one killer from a single infectious disease. Human immunodeficiency virus (HIV) infection is the common risk for re-activation of the latent disease. More than 1.67 million TB deaths were estimated in 2016 around the world. Most of the deaths (78%) were in HIV-positive cases. About 10.4 million TB cases were reported and most of the cases were in Africa including Ethiopia (74%). Ethiopia was ranked as 10^th^ among the 22 high-TB-burden, 26^th^ among the high multidrug resistant-TB-burden, and 13^th^ among the 41 high-TB/HIV-burden countries in the world [1,5,6].

Weakness, weight loss, fever, sweating, cough, and swellings are among the clinical manifestations of tuberculosis. It is a curable and preventable infectious disease. Early diagnosis, effective treatment and timely identification of rifampin resistance are crucial for controlling and eventual elimination of the disease. It is diagnosed by rapid molecular tests, sputum smear microscopy and culture-based methods [4,7].

The control and elimination of TB is currently challenged by the development and spread of microbial resistance to effective, cheap and safe antibiotics. Anti-TB drug resistance is a major medical and public health concern in both developed and developing nations. About 600,000 new TB cases were developed resistance to rifampicin of which 82% had multidrug-resistant TB in 2016. Drug-resistant bacilli strains pose a major risk to the health of every one. It requires longer, complex, expensive and toxic treatment options. In addition, the treatment success rate is much lower. Inadequate dosing and incomplete regimens as well as the ability of the bacilli to develop resistance have fueled the rise of multidrug-resistant TB [1,6,8–13].

High resistance rates of TB are often correlated to the absence of properly implemented control programs including directly observed treatment, short course (DOTS) schemes. In 1994, the DOTS strategy used to control TB was launched by World Health Organization (WHO). The strategy was aimed to detect 70% of infectious cases and cure 85% of them. This was expected to interrupt disease transmission and reduce the number and period of infectiousness. Ethiopia implemented DOTS strategy as pilot programmes in Arsi and Bale zones since 1997. Currently, the DOTS strategy has been subsequently scaled up throughout the country and implemented at national level [4,14,15].

The performance of TB control program is measured by its treatment outcomes as recommended by WHO in 2013. Five exclusive categories of TB treatment outcomes including cured, treatment completed, treatment failed, died and defaulted were used as a standard for global TB data collection and assessment of its treatment performance. The percentage of TB cases that were cured and completed treatment collectively had successful treatment outcome while those with failed treatment, defaulted and died had unsuccessful treatment outcome out of all. Monitoring the treatment outcome of TB is indispensable to appraise the effectiveness and improvement of its intervention and to identify potential barriers for its control [14,16]. However, the performances of TB control program as well as its treatment outcomes have not yet studied in three health centers in East Wollega Zone, Western Ethiopia. Therefore, the aim of the current study was to assess poor treatment outcomes and its determinants among TB patients in selected health facilities in East Wollega zone, Western Ethiopia.

## Methods and materials

### Study area and periods

The study was conducted in three health facilities (Uke health center, Anger Gute health center and Sibu Sire health center) in East Wollega zone, Oromia regional state, Western Ethiopia. These health facilities were purposively selected. The people lived in these areas are highly mobile because the area is the main center for agricultural productivity of the country. Such practices might have a negative impact on TB treatment outcomes. The data was collected from January to March 2017.

### Study design

A five-year retrospective cross-sectional study design was employed to extract the required data from TB patient medical records in the aforementioned health institutions.

### Population

#### Source population

The source population was all TB patients who were treated with anti-TB drugs in the aforementioned health centers.

#### Study population

The study population was all TB patients who were treated with anti-TB drugs from 2012 to 2016 and had treatment outcomes in the aforementioned health centers and fulfilled the selection criteria.

### Variables

The dependent variable was treatment outcome (successful/unsuccessful) while the independent variables were socio-demographic characteristics (sex, age, weight and residence) and clinical characteristics (type of TB, treatment history and HIV status).

### Selection criteria

All TB cases that were registered and treated with anti-TB drugs from 2012 to 2016 and had treatment outcomes were included in the study, whereas registries with missed treatment outcomes, incomplete patient characteristics, and transferred out cases were excluded from the study.

### Data collection

Patient data, including year of treatment, age, sex, weight, residence, TB type, HIV status, treatment history, and treatment outcomes, were extracted from TB patient medical records using a well-structured checklist. Data were collected by experienced nursing staffs working in each TB treatment unit. Data collectors were trained and collected under the strict supervision by the principal investigators.

### Data analysis

Data was entered and analyzed using SPSS version 20. Descriptive statistics was used to generate and summarize frequencies. Univariate and multivariate logistic regression analysis were used to associate the potential determinants of poor treatment outcomes.

### Ethical considerations

Ethical approval was obtained by ethical approval committee (members were Worku Dechassa, Hylemariam Mihretie, Getu Bayissa, and Biniam Paulos) of College of Health Sciences, Wollega University. Letter of permission was written to East Wollega Zone Health Office and to the respective health center administrators to access patients’ data. All data were fully anonymized before access by the researchers. Confidentiality was kept throughout the study. The collected data were used for the intended purpose only.

### Operational definitions

The following term/s used in the study were operationally defined according to:

**Cured.** A pulmonary TB patient with bacteriologically confirmed TB at the beginning of treatment who was smear- or culture-negative in the last month of treatment and on at least one previous occasion.

**Treatment completed.** A TB patient who completed treatment without evidence of failure but with no record to show that sputum smear or culture results in the last month of treatment and on at least one previous occasion were negative, either because tests were not done or because results are unavailable.

**Treatment failed.** A TB patient whose sputum smear or culture is positive at month 5 or later during treatment.

**Defaulted.** A TB patient whose treatment was interrupted for two consecutive months or more.

**Died.** A TB patient who dies for any reason before starting or during the course of treatment.

**Treatment success.** The sum of cured and treatment completed.

**Treatment un-success.** The sum of treatment failed, defaulted and died.

**New TB patients** have not previously been treated for TB and are now diagnosed and started the current treatment.

**Retreated TB patients** have previously been treated for TB and are now diagnosed and started the current treatment with a recurrent episode of TB.

## Results

### Demographic characteristics

**Fig 1:**
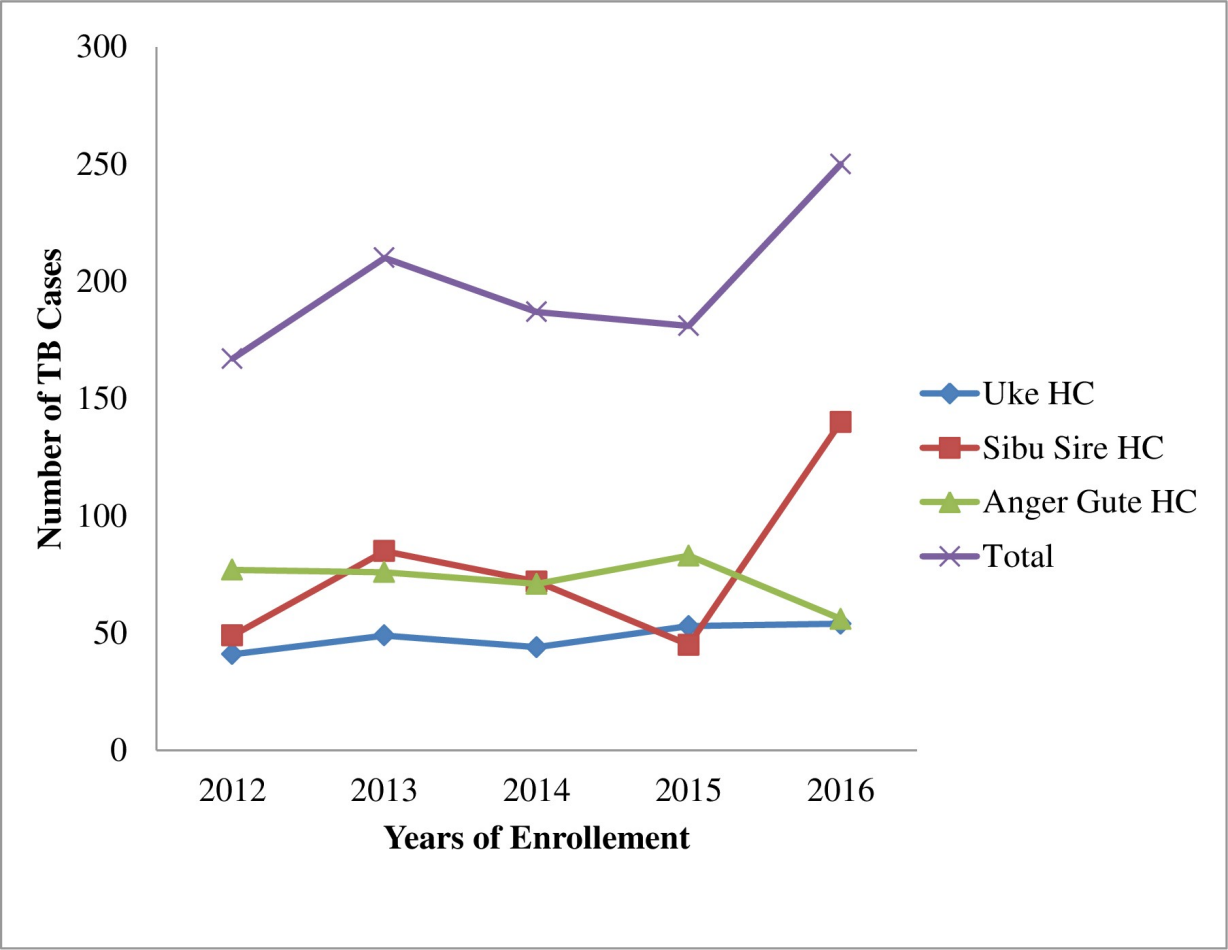
The case incidence of TB patients in three health centers in East Wollega from 2012 to 2016.HC,health center.

During the five-year treatment periods, only 995 TB patients had documented treatment outcomes. Majorities of these patients were males (58.9%), within the age group of 25 to 34 years (25.4%), below the average weight (54.2%) and lived in rural areas (58%) as shown in Table 1. The case incidence of the disease was increased from 2012 to 2014 and decreased from 2014 to 2015 and increased from 2015 to 2016 enrollment years (Fig 1).

**Table 1:** Demographic characteristics of TB patients at three TB treatment centers in East Wollega from 2012 to 2016.

Characteristics		Frequency	Percent	
**Center of treatment**	Sibu Sire health center	391	39.3	
	Anger Gute health center	363	36.	
	Uke health center	241	24.2	
**Sex**	Male	586	58.9	
	Female	409	41.1	
**Age (in years)**	0–14	121	12.2	
	15–24	220	22.1	
	25–34	253	25.4	
	35–44	149	15.0	
	45–54	143	14.4	
	55–64	69	6.9	
	≥65	40	4.0	
	Mean±SD*	31.9±16.3		
**Weight (in kg)**	<45.2	539		54.2
	≥45.2	456		45.8
	Mean±SD*	45.2±12.5		
**Residence**	Rural	577		58.0
	Urban	418		42.0

*SD, standard deviation.

### Clinical characteristics

**Fig 2:**
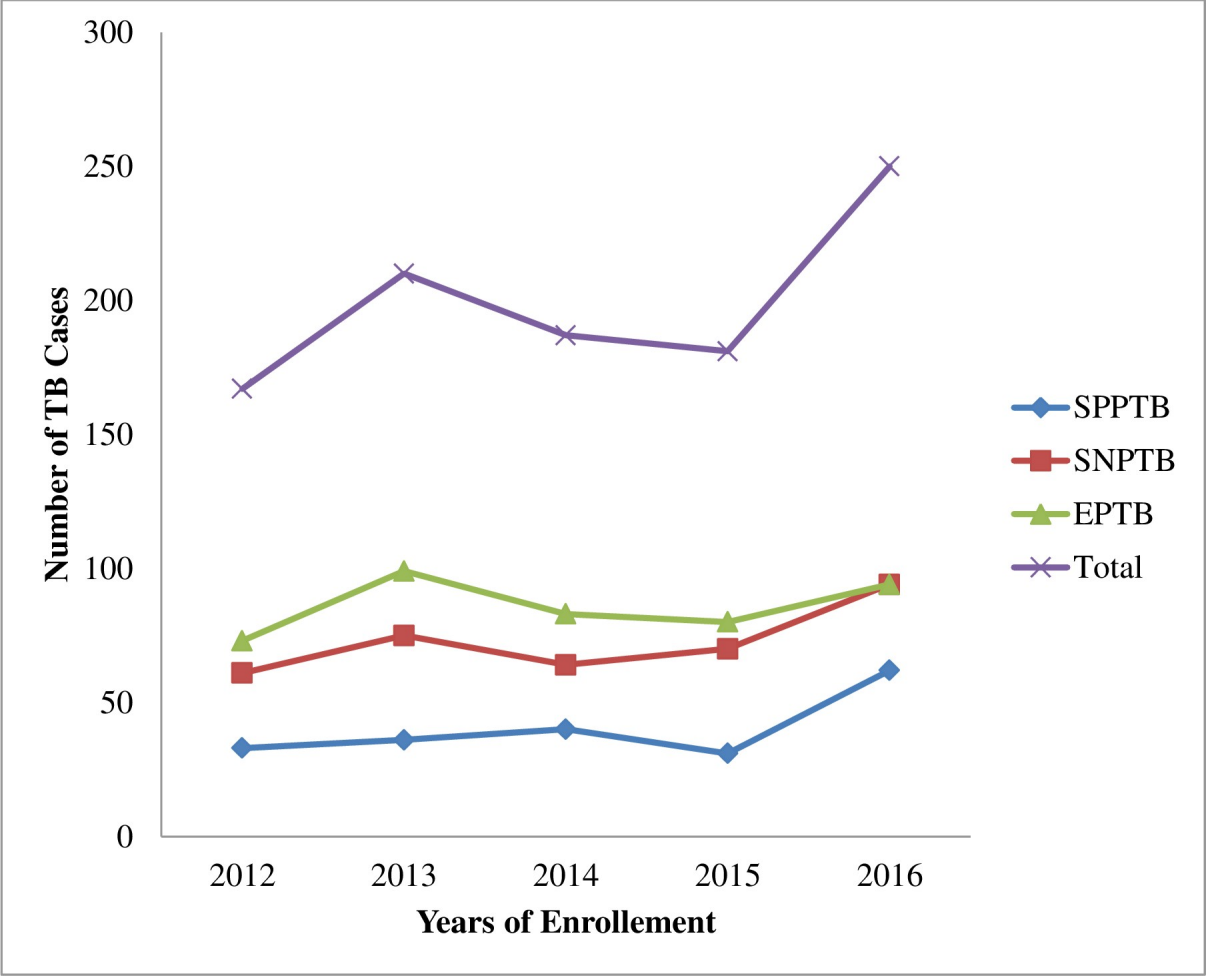
Trends of TB at three TB treatment centers in East Wollega from 2012 to 2016. SPPTB,sputum positive pulmonary tuberculosis; SNPTB,sputum negative pulmonary tuberculosis; EPTB,extrapulmonary tuberculosis.

From 995 cases, most of the patients (95.7%) were new TB patients and 6.8% were co-infected with HIV. The predominant form of TB was extrapulmonary TB (43.1%) as shown in Table 2. The trends of various types of TB were summarized in (Fig 2).

**Table 2:** Clinical characteristics of TB patients at three TB treatment centers in East Wollega from 2012 to 2016.

Characteristics		Frequency	Percent
**Treatment history**	New	952	95.7
	Re-treated	38	3.8
	Unknown	5	0.5
**HIV status**	Positive	68	6.8
	Negative	889	89.3
	Unknown	38	3.8
Tuberculosis type*	SPPTB	202	20.3
	SNPTB	364	36.6
	EPTB	429	43.1

*SPPTB, sputum positive pulmonary tuberculosis; SNPTB, sputum negative pulmonary tuberculosis; EPTB, extrapulmonary tuberculosis.

### Treatment outcomes

**Fig 3:**
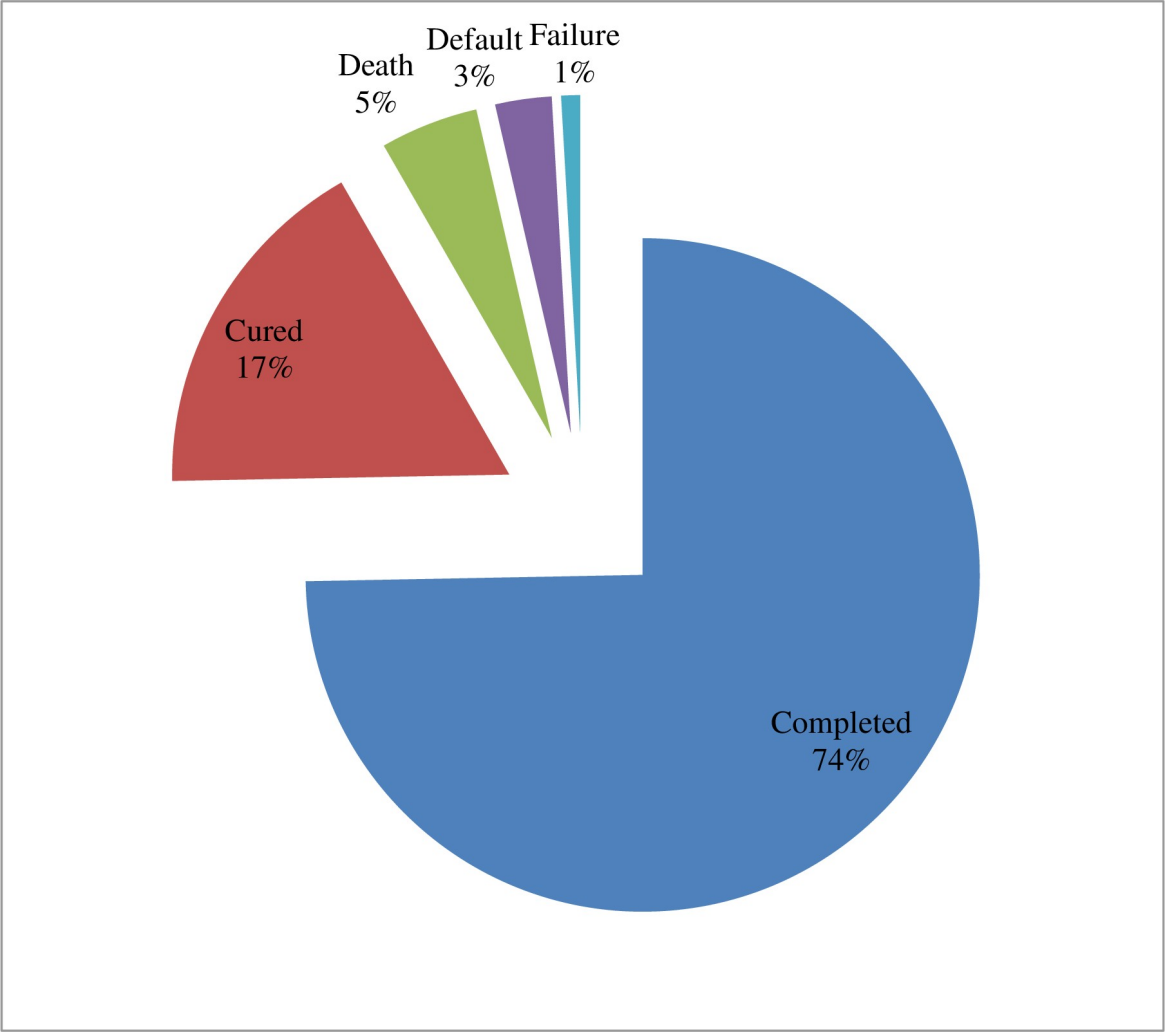
Treatment outcomes of TB patients at three TB treatment centers in East Wollega from 2012 to 2016.

Nearly three-fourth of TB patients (743, 74.7%) had completed their treatment while 171 (17.2%) patients were cured. Twenty nine (2.9%), 48 (4.8%), and 4 (0.4%) patients were defaulted, died, and treatment failed, respectively (Fig 3).

**Fig 4:**
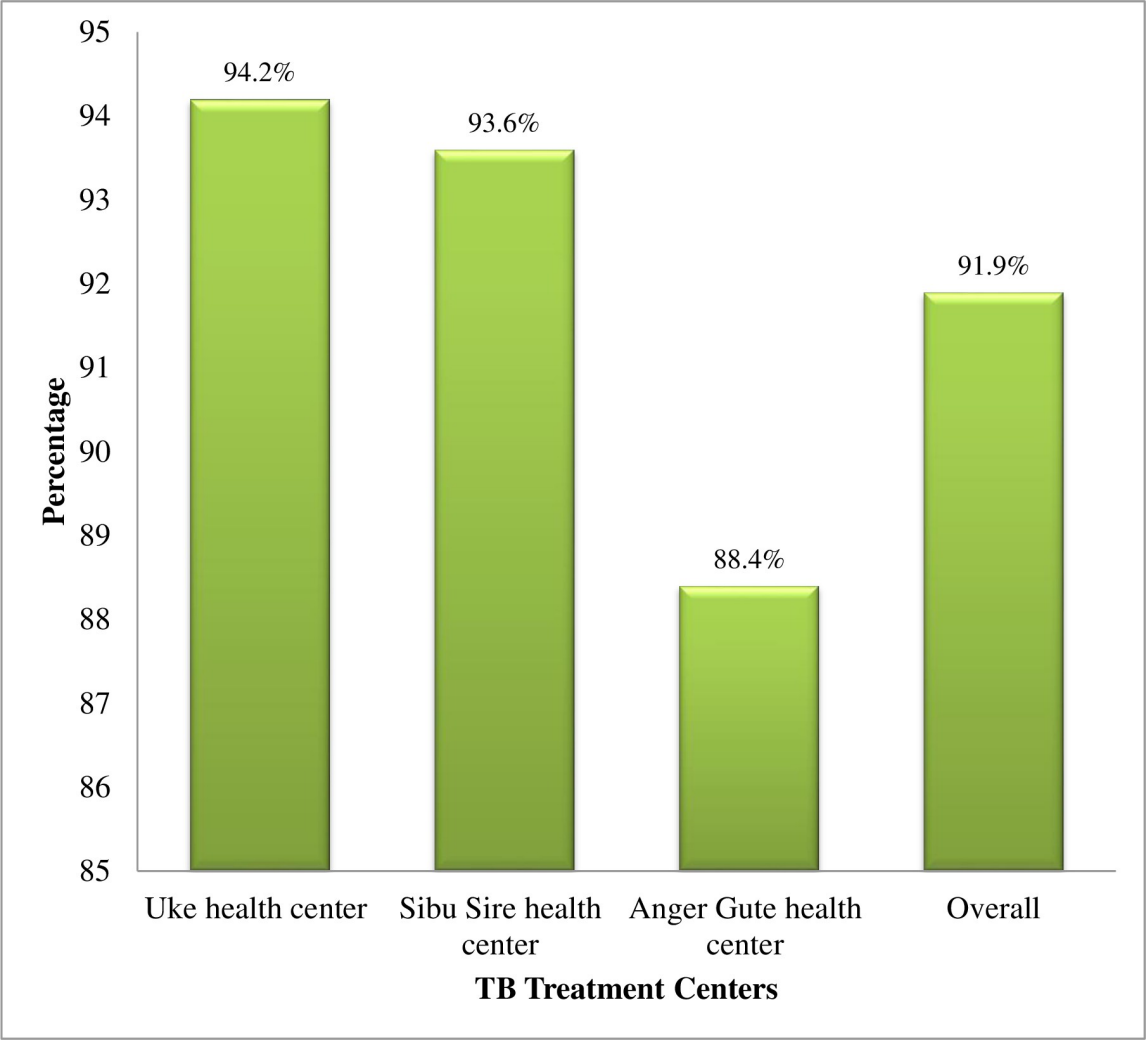
Treatment success outcomes of TB patients at three TB treatment centers in East Wollega from 2012 to 2016.

The overall treatment success rate was 91.9%. Treatment success rate was varied across treatment centers. Higher success rate was observed in Uke health center followed by Sibu Sire and Anger Gute health centers (P = 0.011) (Fig 4). Treatment success rate was higher in female patients (94.1%) compared to males (90.3%) (P = 0.029); urban residents (94%) compared to rural ones (90.3%) (P = 0.034); new TB cases (92.3%) compared with retreated ones (81.6%) (P = 0.037); and HIV-negative patients (92.8%) compared with HIV-positive (88.2%) and unknown status (76.3%) (P = 0.001).

The overall death rate was 4.8%. The death rate was higher in Anger Gute health center (6.6%) compared to Uke health center (2.5%) (P = 0.014); older age (7.7%) compared to age of 25 to 34 years (3.6%) (P = 0.024); male cases (5.8%) compared to females (3.4%) (P = 0.042); retreated cases (10.5%) compared to new cases (4.6%) (P = 0.012); and smear-negative pulmonary TB cases (5.5%) compared to smear-positive ones (3.5%) (P = 0.000).

The overall defaulter rate was 2.9%. The defaulter rate was higher in Anger Gute health center (4.7%) compared to Sibu Sire health center (1.3%) (P = 0.014); older age (8.7%) compared to age of 25 to 34 years (2%) (P = 0.024); male cases (3.4%) compared to females (2.2%) (P = 0.042); retreated cases (5.3%) compared to new cases (2.7%) (P = 0.012); and smear-negative pulmonary TB cases (4.4%) compared to smear-positives (1%) (P = 0.000).

The overall treatment failure rate was 0.4%. The failure rate was higher in male cases (0.5%) compared to females (0.2%) (P = 0.042); retreated cases (2.6%) compared to new cases (0.3%) (P = 0.012); and HIV-negative TB cases (0.4%) compared to HIV-positive ones (0%) (P = 0.005).

### Trends in treatment outcomes

**Fig 5:**
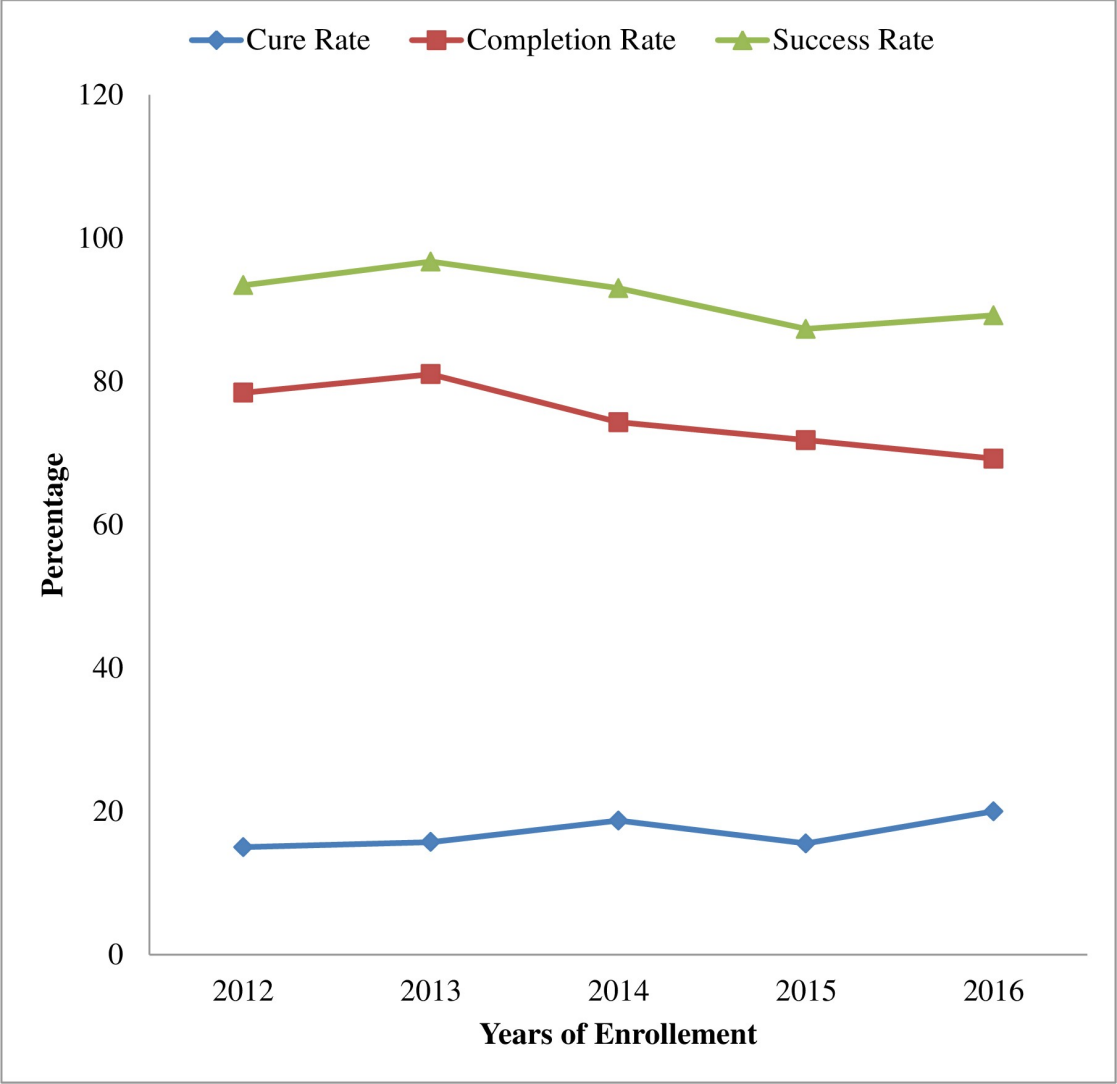
Trends of treatment success rates of TB patients at three TB treatment centers in East Wollega from 2012 to 2016.

The trend in treatment success rate of all TB patients increased from 93.4% in 2012 to 96.7% in 2013 then steadily decreased to 87.3% from 2014 to 2015 and then increased to 89.2% in 2016 (Fig 5).

**Fig 6:**
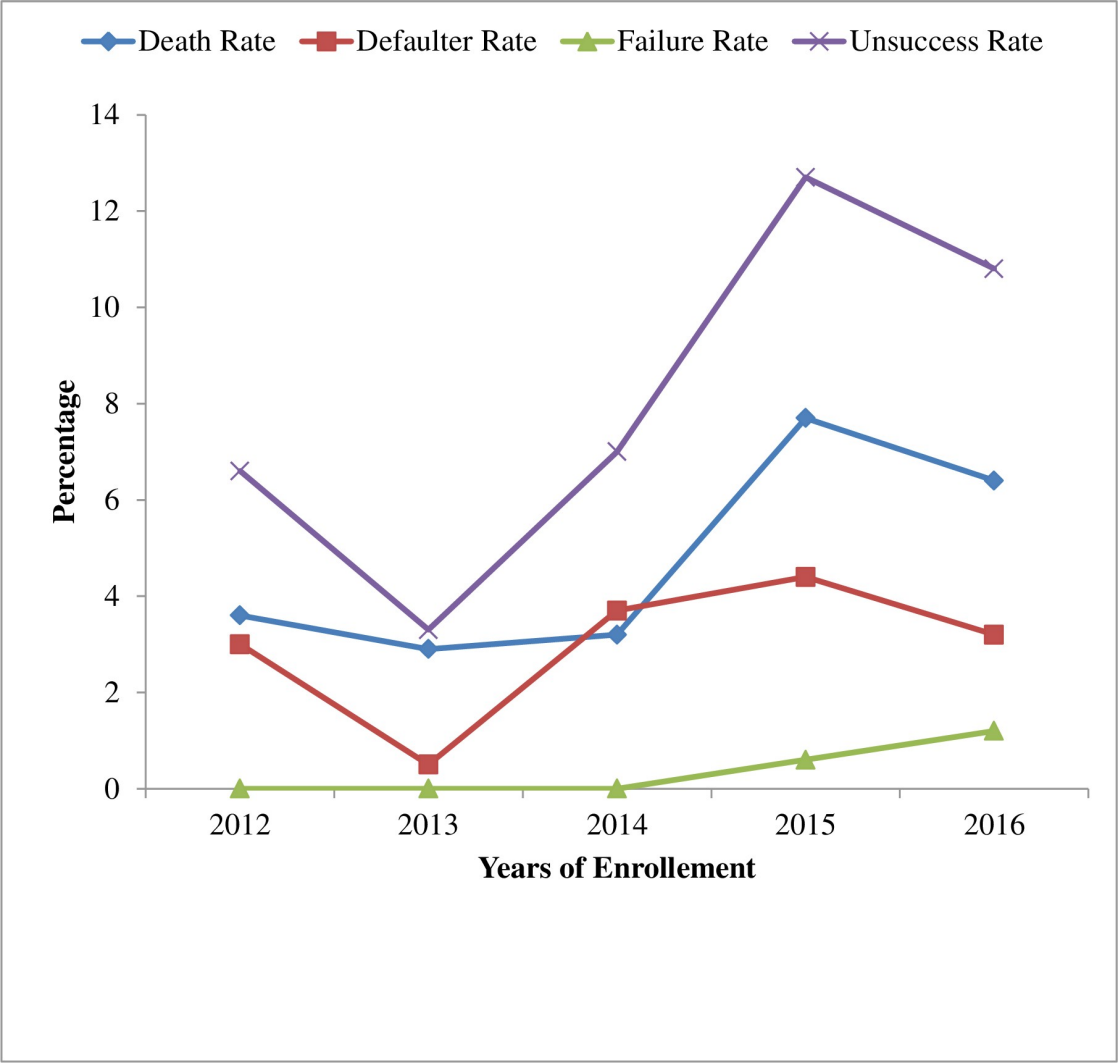
Trends of treatment unsuccessful rates of TB patients at three TB treatment centers in East Wollega from 2012 to 2016.

The death rate decreased from 3.6% in 2012 to 2.9% in 2013 then increased from 3.2% to 7.7% from 2014 to 2015 and again decreased to 6.4% in 2016. The defaulter rate decreased from 3% in 2012 to 0.5% in 2013 then increased to 4.4% from 2014 to 2015 and again decreased into 3.2% in 2016. Treatment failure rate increased from 0.6% in 2015 to 1.2% in 2016. Generally, unfavorable treatment success rate were increased between 2013 and 2015 but started to fall after 2016 (Fig 6).

### Determinants of unsuccessful treatment outcomes

Tuberculosis patients who were treated in Anger Gute health center (adjusted odds ratio (AOR): 2.27; 95% confidence interval (CI): 1.18–4.38; P = 0.015); male (AOR: 1.81; 95% CI: 1.06–3.10; P = 0.031); lived in rural areas (AOR: 1.73; 95% CI: 1.02–2.91; P = 0.041); previously treated (AOR: 2.72; 95% CI: 1.16–6.39; P = 0.022); and unknown status in HIV co-infection (AOR: 4.56; 95% CI: 1.98–10.50; P = 0.000) were at higher risk of unsuccessful treatment outcomes while years of enrollment, age, weight, and tuberculosis type were not determinants of unsuccessful treatment outcomes as shown in Table 3.

**Table 3:** Socio-demographic and clinical variables associated with unsuccessful treatment outcome at three TB treatment centers in East Wollega from 2012 to 2016.

Variables		Treatment outcomes		Crude OR [95% CI]	Adjusted OR [95% CI]
		Successful (%)	Unsuccessful (%)		
**Treatment center**	Uke health center	227 (22.8)	14 (1.4)	1	1
	Sibu Sire health center	366 (36.8)	25 (2.5)	1.11 [0.56–2.18]	1.30 [0.64–2.64]
	Anger Gute health center	321 (32.3)	42 (4.2)	2.12 [1.13–3.98]*	2.27 [1.18–4.38]*
**Age group (years)**	0–14	110 (11.1)	11 (1.1)	1.59 [0.71–3.57]	1.65 [0.67–4.10]
	15–24	204 (20.5)	16 (1.6)	1.24 [0.60–2.58]	1.21[0.57–2.54]
	25–34	238 (23.9)	15 (1.5)	1	1
	35–44	138 (13.9)	11 (1.1)	1.27 [0.57–2.83]	1.10 [0.48–2.53]
	45–54	129 (13.0)	14 (1.4)	1.72 [0.81–3.68]	1.19 [0.53–2.66]
	55–64	60 (6.0)	9 (0.9)	2.38 [0.99–5.70]	1.76 [0.70–4.38]
	≥65	35 (3.5)	5 (0.5)	2.27 [0.78–6.63]	1.53 [0.49–4.77]
**Weight (kg)**	<Average weight	494 (49.6)	45 (4.5)	0.94 [0.60–1.49]	1.01 [0.58–1.56]
	≥Average weight	420 (42.2)	36 (3.6)	1	1
**Sex**	Male	529 (53.2)	57 (5.7)	1.73 [1.05–2.84]*	1.81 [1.06–3.10]*
	Female	385 (38.7)	24 (2.4)	1	1
**Residence**	Urban	393 (39.5)	25 (2.5)	1	1
	Rural	521 (52.4)	56 (5.6)	1.69 [1.04–2.76]*	1.73 [1.02–2.91]*
**Treatment history**	New	879 (88.3)	73 (7.3)	1	1
	Retreated	31 (3.1)	7 (0.7)	2.72 [1.16–6.39]*	2.43 [0.96–6.19]
	Unknown	4 (0.4)	1 (0.1)	3.01 [0.33–27.28]	3.37 [0.35–32.79]
**TB type**	Smear-positive	189 (19.0)	13 (1.3)	1	1
	Smear-negative	328 (33.0)	36 (3.6)	1.60 [0.83–3.08]	1.41 [0.70–2.83]
	Extrapulmonary	397 (39.9)	32 (3.2)	1.17 [0.60–2.28]	1.09 [0.54–2.23]
**HIV status**	Negative	825 (82.9)	64 (6.4)	1	1
	Positive	60 (6.0)	8 (0.8)	1.72 [0.79–3.75]	1.16 [0.95–4.90]
	Unknown	29 (2.9)	9 (0.9)	4.00 [1.82–8.81]*	4.56 [1.98–10.50]*

*P-value< 0.05.

## Discussion

The implementation and expansion of DOTS strategy have been substantially improved the success rate of TB treatment outcomes. However, the strategy is being challenged by poor treatment outcomes especially in low and middle-income countries. Therefore, routine monitoring of TB treatment performances and analysis of determinants responsible for poor treatment outcomes are vital to prevent further adverse outcomes.

Of 995 patients with documented treatment outcomes, more than half of them (58.9%) were males. This was similar with reports by WHO [1] and in southern Ethiopia [17]. This might be due to biological differences, difference in societal roles and access to health facilities. Majority of TB cases were within productive age groups (83.8%), which was comparable with other reports in southern Ethiopia [17] and central Ethiopia [18]. This might reflect the negative impact of the disease on socio-economic condition of the society. Extrapulmonary TB constituted the common form of TB which was in line with other report in central Ethiopia [18].

The overall treatment success rate was 91.9%, which was exceeded to the WHO target [1]. This was comparable to other findings in northeast Ethiopia (90.1%) [19] and Northwest Ethiopia (94%, 94.4%) [20,21]. But the current finding was higher than with other reports in north Ethiopia (60.1%, 79.3%) [22,23], south Ethiopia (74%) [17], northwest Ethiopia (80.5%, 87.8%) [24,25], central Ethiopia (82.7%, 83.6%) [18,26] and east Ethiopia (83.9%) [27]. This disagreement might be differences in handling of transfer out cases, socio-economic characteristics of patients, geographic setting, sample size, study period, HIV co-infection, DOTS performance, and coordination of HIV and TB activities. However, a declining trend of treatment success rate was observed over the treatment periods.

Treatment success rate was significantly varied across treatment centers. Unfavorable treatment outcome was observed in Anger Gute health center than others (AOR: 2.27; 95% CI: 1.18–4.38; P = 0.015). Similar treatment success variation across treatment centers also reported in central Ethiopia [18] and east Ethiopia [26]. The possible reasons might be differences in human resources and their productivity, workload and heterogeneity of service provision.

Male patients were more likely to have poor treatment outcome when compared to females (AOR: 1.81; 95% CI: 1.06–3.10; P = 0.031). A similar finding was reported elsewhere in north Ethiopia [28], northwest Ethiopia [29], southern Ethiopia [30] and worldwide [31]. Being highly exposed to cigarette smoking, alcohol consumption and HIV prevalence and travelling a long distance for economic reasons might contribute to poor treatment success rate in men.

Patients from rural areas were more likely to have poor treatment outcome than urban dwellers (AOR = 1.173; 95% CI: 1.02–2.91; P = 0.041). This was in agreement with other reports elsewhere in northwest Ethiopia [32,33], southern Ethiopia [30] and southwest Ethiopia [34]. Lower awareness and information about TB, HIV and their treatments, long distance between their homes and treatment centers, fear of stigma and discrimination, and poor socio-economic status might contribute to lower treatment success rate in rural patients.

Previously treated patients were more likely to have poor treatment outcome than newly treated ones (AOR = 1.72; 95% CI: 1.16–6.39; P = 0.022). This was in line with previous reports in northwest Ethiopia [22,29], east Ethiopia [26], central Ethiopia [30] and southwest Ethiopia [34]. Prior suboptimal therapy, poor compliance and high prevalence of multidrug resistant-TB might contribute the observed variation. Due to limited access to culture and drug sensitivity test services in Ethiopia, the extent of multidrug resistant-TB among re-treatment cases are mostly unknown.

Being unknown status about HIV-coinfection had also increased the risk of unsuccessful treatment outcome (AOR: 4.56; 95% CI: 1.98–10.50; P = 0.000). A similar finding was reported in northwest Ethiopia [22,24] and central Ethiopia [30]. The possible reason could be if these patients were HIV positive and not initiate antiretroviral therapy timely, the consequence were bad and contributory factor for unsuccessful treatment outcome. Other comorbidities such as diabetes and chronic conditions might be the other contributory reasons.

More than half of patients with unsuccessful treatment outcomes (58.5%) were died. This was in line with other reports in east Ethiopia [27] and south Ethiopia [35] but lower than previous studies [17,26,32] in Ethiopia. Re-treated patients (10.5%) were more likely to die than newer patients (4.6%). Patients of unknown HIV status (10.5%) were more likely to die than HIV negative cases (4.5%). Patients lived in rural areas (5.9%) were also more likely to die than urban residents (3.3%).

The overall defaulter rate was 1.6% which was lower than a target recommended by WHO (≤3%) [36]. The current finding was similar to a report in northeast Ethiopia [19] but much lower than other findings [17,23,26,32,35,37,38] in Ethiopia. However, there was still a defaulter rate variation across the study settings and treatment periods. Previously retreated patients (5.3%) were more likely to default than newer cases (2.7%). Patients of unknown HIV co-infection status (13.2%) were more likely to default than HIV negative cases (2.5%). This was in agreement with a report in northeast Ethiopia [19].

The overall treatment failure rate was 0.5% which was comparable with other studies [18,20,23,27,29,32,35] but lower than others [19,20,26,37] in Ethiopia. Retreated patients (2.6%) were more likely to fail than new cases (0.3%). Previous exposure to anti-TB drugs might increase the risk of developing multidrug resistant-TB. Hence, strengthening the capacity of TB diagnostic facilities for early detection and management of multidrug resistant-TB cases is important.

### Limitation of the study

The major limitation of the study was related to the use of secondary data. We have not had access to the full range of the socio-demographic, socio-economic and clinical variables including alcohol consumption, cigarette smoking, and comorbidity in the medical records during the review process. The historical data used in this study was originally collected primarily for reporting purposes. The weakness of this type of data is well known, especially, in low income countries, where the qualities of reports are often poor. In addition, data analysis from retrospective reviews of patient records might affect the validity of the result.

## Conclusion

Tuberculosis treatment success rate was exceeded the 90% treatment success rate target as set by WHO. However, the success rate was consistently varied across the study settings and treatment periods. It was consecutively declined with treatment periods. Special attention is therefore required for patients with high risk of unsuccessful treatment outcome including those patients treated in Anger Gute health centre, came from rural areas, treated again and with unknown HIV status need strict follow up throughout their treatment periods. Supportive supervision should be initiated and strengthened to maintain the high treatment success rate as well as to reverse the current declining treatment success rate of tuberculosis.

## Supporting information

S1 Table Demographic and clinical characteristics of tuberculosis patients treated from 2012 to 2016 in three health facilities in East Wollega, Western Ethiopia. UHC, Uke health center; SSHC, Sibu Sire health center; AGHC, Anger Gute health center; M, male; F, female; U, urban; R, rural; RT, Re-treated; SP, smear-positive; SN, smear-negative; EP, extrapulmonary; P, positive, N, negative; NA, not applicable, NE, not evaluated; NT, not tested; C, cured; CT, completed treatment; D, defaulted; Di, died.
(PDF)
