# Supplementary material for: Poor treatment outcomes and its determinants among tuberculosis patients in selected health facilities in East Wollega, Western Ethiopia
Source: PLoS One. 2018 Oct 26;13(10):e0206227. doi: 10.1371/journal.pone.0206227 (PMC6203372; doi:10.1371/journal.pone.0206227)
Supplement: S1 Table — UHC, Uke health center; SSHC, Sibu Sire health center; AGHC, Anger Gute health center; M, male; F, female; U, urban; R, rural; RT, Re-treated; SP, smear-positive; SN, smear-negative; EP, extrapulmonary; P, positive, N, negative; NA, not applicable, NE, not evaluated; NT, not tested; C, cured; CT, completed treatment; D, defaulted; Di, died. (PDF) [file pone.0206227.s001.pdf]

S1 Table. Demographic and clinical characteristics of tuberculosis patients treated from 2012 to 2016 in three health facilities in East Wollega, Western Ethiopia.

S. No	Center of treatment	Card No	Age (yrs)	Weight (Kg)	Sex	Residence	Treatment Years	Treatment history	Tuberculosis type	Sputum smear results at 2 months	Sputum smear results at 5 months	Sputum smear results at 7 months	HIV status	Treatment outcomes
1.			25	50	M	U	2012	New	EP	NA	NA	NA		D
2.			35	51	F	U	2012	New	SP	N	N	N		C
3.			22	53	M	U	2012	New	EP	NA	NA	NA		CT
4.			65	40	M	R	2012	New	EP	NA	NA	NA		CT
5.			32	55	M	U	2012	New	SN	NA	NA	NA		CT
6.			5	16	F	R	2012	New	SN	NA	NA	NA		CT
7.			33	51	F	R	2012	New	EP	NA	NA	NA		CT
8.			31	53.8	F	U	2012	New	EP	NA	NA	NA		CT
9.			37	51	M	U	2012	New	SN	NA	NA	NA		CT
10.			30	49	M	U	2012	New	EP	NA	NA	NA		CT
11.			14	29	M	R	2012	New	EP	NA	NA	NA		CT
12.			28	50	F	R	2012	New	EP	NA	NA	NA		CT
13.			33	55.2	M	R	2012	New	SN	NA	NA	NA		CT
14.			48	61	M	U	2012	New	SN	NA	NA	NA		CT
15.			60	49	M	R	2012	RT	SP	N	N	N		C
16.			26	48	M	R	2012	New	SP	N	NT	NT		D
17.			25	56	M	U	2012	New	EP	NA	NA	NA		CT
18.			52	53	M	R	2012	New	SP	N	N	N		C
19.			37	40	M	R	2012	New	SN	NA	NA	NA		CT
20.			40	43	M	R	2012	New	SP	N	N	NT		CT
21.			55	48	M	R	2012	New	SN	NA	NA	NA		Di
22.			12	17	F	R	2012	New	SP	N	N	NT		CT

23.			16	50	F	R	2012	New	EP	NA	NA	NA		CT
24.			29	40.7	F	R	2012	New	EP	NA	NA	NA		CT
25.			25	48	F	R	2012	New	EP	NA	NA	NA		CT
26.			45	40	M	R	2012	New	SN	NA	NA	NA		CT
27.			50	42.5	F	U	2012	New	EP	NA	NA	NA		CT
28.			17	50	F	R	2012	New	SN	NA	NA	NA		CT
29.			30	57	M	R	2012	New	SN	NA	NA	NA		CT
30.			20	40	F	R	2012	New	SN	NA	NA	NA		CT
31.			35	44	F	R	2012	New	SN	NA	NA	NA		CT
32.			23	50	M	U	2012	New	EP	NA	NA	NA		CT
33.			25	53	F	U	2012	New	EP	NA	NA	NA		CT
34.			25	55	M	R	2012	New	EP	NA	NA	NA		CT
35.			15	35	F	R	2012	New	SP	N	N	N		C
36.			60	45	M	R	2012	RT	SN	NA	NA	NA		CT
37.			15	41	F	U	2012	New	SN	NA	NA	NA		CT
38.			30	44	M	R	2012	New	SP	N	N	N		C
39.			20	58	M	R	2012	New	EP	NA	NA	NA		CT
40.			18	7	M	R	2012	New	SP	N	N	N		C
41.			22	50	F	U	2013	New	EP	NA	NA	NA		CT
42.			28	3	M	R	2013	New	SN	NA	NA	NA		CT
43.			20	50	M	R	2013	New	SN	NA	NA	NA		CT
44.			25	41	F	U	2013	New	EP	NA	NA	NA		Di
45.			26	54	M	R	2013	New	EP	NA	NA	NA		CT
46.			2	9	F	U	2013	New	EP	NA	NA	NA		CT
47.			30	49	M	R	2013	New	EP	NA	NA	NA		CT
48.			65	51	M	R	2013	New	EP	NA	NA	NA		CT
49.			25	53	M	R	2013	RT	SP	N	N	N		C
50.			14	42	F	R	2013	New	EP	NA	NA	NA		CT
51.			40	38	F	R	2013	New	SN	NA	NA	NA		CT
52.			45	50	M	R	2013	New	EP	NA	NA	NA		CT
53.			26	56	M	R	2013	New	SN	NA	NA	NA		CT

54.			24	50	M	U	2013	New	SP	N	N	N		C
55.			34	60	F	U	2013	New	EP	NA	NA	NA		CT
56.			45	60	M	R	2013	New	EP	NA	NA	NA		CT
57.			26	63	M	U	2013	New	EP	NA	NA	NA		CT
58.			31	47	M	R	2013	New	EP	NA	NA	NA		CT
59.			45	53	M	R	2013	New	SN	NA	NA	NA		CT
60.			38	46.5	M	R	2013	New	EP	NA	NA	NA		CT
61.			22	40	F	U	2013	New	SN	NA	NA	NA		CT
62.			45	45	M	R	2013	New	SN	NA	NA	NA		CT
63.			28	46	M	R	2013	New	EP	NA	NA	NA		CT
64.			3	15	F	U	2013	New	EP	NA	NA	NA		CT
65.			28	55	M	R	2013	New	SN	NA	NA	NA		CT
66.			4	14	M	U	2013	New	EP	NA	NA	NA		CT
67.			50	46	F	R	2013	New	SP	N	N	N		C
68.			40	53	F	R	2013	New	SN	NA	NA	NA		CT
69.			28	47	M	R	2013	New	SP	N	N	N		C
70.			28	43	F	R	2013	New	SN	NA	NA	NA		CT
71.			30	45	M	R	2013	Uk	SN	NA	NA	NA		CT
72.			25	54	M	U	2013	New	SN	NA	NA	NA		CT
73.			13	32	M	R	2013	New	SN	NA	NA	NA		CT
74.			55	44	M	R	2013	RT	SP	N	N	N		C
75.			41	52	F	R	2013	New	EP	NA	NA	NA		CT
76.			20	43.8	M	R	2013	New	SP	N	N	N		C
77.			20	41	M	R	2013	New	SP	N	N	N		C
78.			3	13.5	M	R	2013	New	SN	NA	NA	NA		CT
79.			28	48	F	R	2013	New	SP	N	N	NT		CT
80.			50	50	M	R	2013	New	SP	N	N	N		C
81.			18	44	M	R	2013	New	SN	NA	NA	NA		CT
82.			50	42	F	R	2013	New	SN	NA	NA	NA		CT
83.			55	48	M	U	2013	New	SN	NA	NA	NA		CT
84.			13	31	M	R	2013	New	EP	NA	NA	NA		CT

85.			28	42.7	F	R	2013	New	SN	NA	NA	NA		CT
86.			40	50	F	0	2013	New	SN	NA	NA	NA		CT
87.			48	43	M	R	2013	RT	SP	N	N	N		C
88.			40	52.4	M	R	2013	New	SP	N	N	N		C
89.			25	48.5	M	R	2013	New	SP	N	N	N		C
90.			7	20	F	R	2014	New	EP	NA	NA	NA		CT
91.			46	47.1	M	R	2014	New	SP	N	N	N		C
92.			45	58	F	U	2014	RT	SP	N	N	N		C
93.			20	60	M	U	2014	New	EP	NA	NA	NA		CT
94.			36	44	F	U	2014	New	SP	N	N	N		C
95.			22	45.5	M	R	2014	New	SN	NA	NA	NA		CT
96.			38	46	M	U	2014	New	SP	N	N	N		C
97.			30	34.8	M	U	2014	New	SP	N	N	N		C
98.			50	35	M	U	2014	New	SN	NA	NA	NA		CT
99.			19	44	F	U	2014	New	EP	NA	NA	NA		CT
100.			50	61.5	M	U	2014	New	SP	N	N	N		C
101.			30	35	F	R	2014	New	SN	NA	NA	NA		CT
102.			25	47	F	R	2014	New	SP	N	N	N		C
103.			13	36	M	R	2014	New	EP	NA	NA	NA		CT
104.			20	48	M	U	2014	New	EP	NA	NA	NA		C
105.			25	47.3	F	U	2014	New	EP	NA	NA	NA		CT
106.			55	50	M	R	2014	New	SN	NA	NA	NA		CT
107.			56	45	M	R	2014	New	EP	NA	NA	NA		CT
108.			25	46	M	R	2014	New	SP	N	N	N		C
109.			23	54	M	R	2014	New	SN	NA	NA	NA		CT
110.			46	50	F	U	2014	New	SN	NA	NA	NA		CT
111.			23	49.6	M	U	2014	New	SP	N	N	N		C
112.			12	28.4	F	R	2014	New	SP	N	N	NT		CT
113.			25	47	F	U	2014	Uk	SN	NA	NA	NA		D
114.			26	47	F	U	2014	New	SP	N	N	N		C
115.			55	40	F	R	2014	New	SN	NA	NA	NA		CT

116.			24	54.7	F	U	2014	New	EP	NA	NA	NA		CT
117.			38	53.2	M	R	2014	New	SP	N	N	N		C
118.			45	44	M	R	2014	New	SN	NA	NA	NA		CT
119.			2	10	M	U	2014	New	EP	NA	NA	NA		CT
120.			33	60	F	R	2014	New	SN	NA	NA	NA		CT
121.			13	40	M	U	2014	New	EP	NA	NA	NA		CT
122.			37	49	F	R	2014	New	SP	N	N	N		C
123.			14	35	M	R	2014	New	EP	NA	NA	NA		CT
124.			3	14	M	R	2014	New	EP	NA	NA	NA		CT
125.			60	55	M	U	2014	New	EP	NA	NA	NA		CT
126.			70	65	M	U	2014	New	SN	NA	NA	NA		CT
127.			14	40	F	R	2012	New	SN	N	N	P		C
128.			50	42	M	R	2014	New	SN	NA	NA	NA		CT
129.			22	50	M	R	2014	New	EP	NA	NA	NA		CT
130.			60	49.7	M	R	2014	New	EP	NA	NA	NA		CT
131.			56	50	M	U	2014	New	SN	NA	NA	NA		CT
132.			40	38	F	R	2014	RT	SP	N	N	N		C
133.			18	26	F	R	2014	New	SN	NA	NA	NA		CT
134.			25	37.4	F	R	2014	New	SN	NA	NA	NA		CT
135.			29	57	M	U	2015	New	SN	NA	NA	NA		CT
136.			42	54	M	R	2015	New	SN	NA	NA	NA		CT
137.			25	50	M	R	2015	New	EP	NA	NA	NA		CT
138.			7	22.3	M	U	2015	New	SN	NA	NA	NA		CT
139.			11	24.4	M	U	2015	New	SN	NA	NA	NA		CT
140.			30	43	M	R	2015	New	SN	NA	NA	NA		CT
141.			55	52	M	R	2015	New	EP	NA	NA	NA		D
142.			30	48	F	R	2015	New	EP	NA	NA	NA		CT
143.			38	68	F	U	2015	New	SN	NA	NA	NA		CT
144.			35	40.8	M	R	2015	RT	SP	N	N	N		C
145.			38	50	M	R	2015	New	EP	NA	NA	NA		CT
146.			55	46	F	R	2015	New	SN	NA	NA	NA		CT

147.			7	19	F	R	2015	New	EP	NA	NA	NA		CT
148.			28	35	F	U	2015	New	SN	NA	NA	NA		CT
149.			2	10.5	F	R	2015	New	SN	NA	NA	NA		CT
150.			18	44.7	M	R	2015	New	SN	NA	NA	NA		CT
151.			50	46.7	M	R	2015	RT	SP	N	N	N		C
152.			40	41	M	R	2015	New	SP	N	N	N		C
153.			25	41	F	U	2015	New	EP	NA	NA	NA		CT
154.			40	58	M	R	2015	New	SP	N	N	N		C
155.			5	11	M	R	2015	New	EP	NA	NA	NA		Di
156.			30	48.3	M	R	2015	New	SN	NA	NA	NA		CT
157.			14	27	M	R	2015	New	SN	NA	NA	NA		CT
158.			7	18	F	R	2015	New	EP	NA	NA	NA		CT
159.			23	41.2	F	R	2015	New	EP	NA	NA	NA		D
160.			60	48.8	M	R	2015	New	SN	NA	NA	NA		CT
161.			18	50	M	R	2015	New	EP	NA	NA	NA		CT
162.			35	53	M	U	2015	New	EP	NA	NA	NA		Di
163.			21	56	M	R	2015	New	SN	NA	NA	NA		CT
164.			19	61	M	R	2015	New	EP	NA	NA	NA		CT
165.			39	51	M	R	2015	New	EP	NA	NA	NA		CT
166.			38	46	F	R	2015	New	EP	NA	NA	NA		CT
167.			54	60	M	R	2015	New	SP	N	N	N		C
168.			24	46.6	F	U	2015	New	SN	NA	NA	NA		CT
169.			22	45	M	R	2015	New	SP	N	N	N		C
170.			22	55	M	R	2015	New	SN	NA	NA	NA		CT
171.			55	46	M	R	2015	New	SN	NA	NA	NA		CT
172.			28	42.8	F	R	2015	New	EP	NA	NA	NA		CT
173.			20	57.7	M	R	2015	New	SN	NA	NA	NA		CT
174.			28	50	M	R	2015	New	EP	NA	NA	NA		CT
175.			22	53.6	F	R	2015	New	EP	NA	NA	NA		CT
176.			35	40	F	R	2015	New	SN	NA	NA	NA		CT
177.			18	45	F	R	2015	New	SN	NA	NA	NA		CT

178.			50	50	M	U	2015	New	SN	NA	NA	NA		CT
179.			18	40	F	U	2015	New	SN	NA	NA	NA		CT
180.			55	41.8	F	R	2015	New	SP	N	N	N		C
181.			19	37	F	R	2015	New	SP	P	NT	P		C
182.			42	65	M	U	2015	New	EP	NA	NA	NA		CT
183.			27	42.3	F	R	2015	New	SN	NA	NA	NA		CT
184.			17	51.2	M	R	2015	New	SP	N	N	N		C
185.			27	61.1	M	U	2015	New	EP	NA	NA	NA		CT
186.			21	57.3	F	U	2015	New	SP	N	N	N		C
187.			20	51	M	R	2015	New	SN	NA	NA	NA		CT
188.			40	46	F	U	2016	New	SP	N	NT	NT		CT
189.			25	55	F	U	2016	New	EP	NA	NA	NA		CT
190.			21	42	F	R	2016	New	SP	N	N	NT		CT
191.			40	40	F	U	2016	New	SN	NA	NA	NA		CT
192.			60	50	M	U	2016	RT	SN	NA	NA	NA		CT
193.			40	50	M	R	2016	New	SP	NT	N	N		C
194.			31	50	M	R	2016	New	SN	NA	NA	NA		CT
195.			27	56	M	U	2016	New	SN	NA	NA	NA		CT
196.			35	34	F	R	2016	New	SP	NT	N	N		C
197.			10	21	F	R	2016	RT	SN	NA	NA	NA		CT
198.			45	50	M	R	2016	RT	SN	NA	NA	NA		CT
199.			50	53	M	R	2016	New	EP	NA	NA	NA		CT
200.			57	56	M	R	2016	RT	SN	NA	NA	NA		CT
201.			25	38	F	R	2016	New	SN	NA	NA	NA		CT
202.			40	46	M	R	2016	New	SP	N	N	NT		TR
203.			1	8.5	F	R	2016	New	SN	NA	NA	NA		CT
204.			27	42	F	R	2016	New	SN	NA	NA	NA		CT
205.			52	65	M	R	2016	New	SN	NA	NA	NA		CT
206.			47	43	M	R	2016	New	SN	NA	NA	NA		CT
207.			38	39	F	R	2016	New	SN	NA	NA	NA		CT
208.			45	65	M	R	2016	New	SN	NA	NA	NA		CT

209.			48	52	M	R	2016	New	SN	NA	NA	NA		CT
210.			35	41	M	R	2016	New	SN	NA	NA	NA		CT
211.			0.5	6.5	M	R	2016	New	SN	NA	NA	NA		D
212.			3	11	F	R	2016	New	SN	NA	NA	NA		D
213.			3	9.6	M	R	2016	New	EP	NA	NA	NA		CT
214.			3	12	F	R	2016	New	EP	NA	NA	NA		CT
215.			38	47	M	R	2016	New	SN	NA	NA	NA		Di
216.			40	38	F	R	2016	New	SN	NA	NA	NA		CT
217.			20	44	F	R	2016	New	SN	NA	NA	NA		CT
218.			25	41	F	U	2016	New	SP	N	NT	N		C
219.			50	42.7	F	R	2016	New	SP	N	N	N		C
220.			17	44	F	U	2016	New	SP	P	N	N		C
221.			20	40	F	U	2016	New	SP	N	N	N		C
222.			31	56	M	R	2016	New	EP	NA	NA	NA		CT
223.			26	56	M	R	2016	New	EP	NA	NA	NA		CT
224.			28	32	M	R	2016	New	SN	NA	NA	NA		CT
225.			20	44	M	R	2016	New	SP	N	N	N		C
226.			25	57	M	R	2016	New	SN	NA	NA	NA		CT
227.			33	53	M	U	2016	New	EP	NA	NA	NA		CT
228.			35	53	F	U	2016	New	SN	NA	NA	NA		CT
229.			23	53	M	U	2016	New	SP	P	N	N		C
230.			11	34	F	R	2016	New	SN	NA	NA	NA		CT
231.			48	50	M	U	2016	New	EP	NA	NA	NA		CT
232.			14	50	M	U	2016	New	EP	NA	NA	NA		CT
233.			25	30.8	F	R	2016	New	EP	NA	NA	NA		CT
234.			60	42	M	U	2016	New	EP	NA	NA	NA		CT
235.			17	39	F	U	2016	New	SN	NA	NA	NA		CT
236.			35	45	F	R	2016	New	SP	N	N	NT		CT
237.			19	50	M	R	2016	New	SN	NA	NA	NA		CT
238.			7	14	M	U	2016	New	SN	NA	NA	NA		Di
239.			55	35	M	U	2016	RT	SN	NA	NA	NA		CT

240.			12	25	F	R	2016	New	EP	NA	NA	NA		CT
241.			50	35	F	R	2016	New	SN	NA	NA	NA		CT
242.			34	54	M	R	2012	New	EP	NA	NA	NA		CT
243.			45	47	M	U	2012	New	SN	NA	NA	NA		CT
244.			24	50	F	R	2012	New	EP	NA	NA	NA		CT
245.			20	53	M	R	2012	New	SN	NA	NA	NA		CT
246.			30	45	M	U	2012	New	SN	NA	NA	NA		CT
247.			34	47	M	R	2012	New	SN	NA	NA	NA		CT
248.			17	43	M	U	2012	New	SP	N	N	N		C
249.			18	19	F	U	2012	New	EP	NA	NA	NA		CT
250.			25	52	M	R	2012	New	EP	NA	NA	NA		CT
251.			45	47	F	R	2012	New	EP	NA	NA	NA		CT
252.			18	43	F	U	2012	New	SN	NA	NA	NA		CT
253.			21	31	F	U	2012	New	EP	NA	NA	NA		CT
254.			50	42	F	U	2012	New	EP	NA	NA	NA		CT
255.			35	44	F	U	2012	New	EP	NA	NA	NA		CT
256.			20	46	M	R	2012	New	EP	NA	NA	NA		CT
257.			25	41	F	R	2012	New	EP	NA	NA	NA		CT
258.			30	60	M	U	2012	New	EP	NA	NA	NA		CT
259.			35	55	F	R	2012	New	SN	NA	NA	NA		CT
260.			60	40	F	R	2012	New	EP	NA	NA	NA		CT
261.			50	40	F	R	2012	New	EP	NA	NA	NA		CT
262.			25	47	M	U	2012	New	SN	NA	NA	NA		CT
263.			37	45	F	U	2012	New	SN	NA	NA	NA		CT
264.			37	45	F	U	2012	New	SN	NA	NA	NA		CT
265.			30	45	M	U	2012	New	SN	NA	NA	NA		CT
266.			30	50	M	R	2012	New	SP	N	N	N		C
267.			21	49	M	R	2012	New	SN	NA	NA	NA		CT
268.			47	38	M	U	2012	New	EP	NA	NA	NA		CT
269.			60	46	M	U	2012	New	SN	NA	NA	NA		CT
270.			21	42	M	R	2012	New	SP	N	N	N		C

271.			29	55	M	U	2012	New	EP	NA	NA	NA		CT
272.			50	50	F	U	2012	New	SN	NA	NA	NA		CT
273.			35	50	M	R	2012	New	EP	NA	NA	NA		CT
274.			38	60	M	R	2012	New	SP	N	N	N		CT
275.			18	33	F	R	2012	New	SP	N	N	N		C
276.			50	45	M	R	2012	New	SN	NA	NA	NA		CT
277.			67	45	F	R	2012	New	SN	NA	NA	NA		CT
278.			28	45	M	R	2012	New	SP	N	N	N		C
279.			19	60	M	U	2012	New	EP	NA	NA	NA		CT
280.			25	55	M	U	2012	New	EP	NA	NA	NA		CT
281.			47	39	M	U	2012	New	EP	NA	NA	NA		CT
282.			15	30	M	R	2012	New	SN	NA	NA	NA		CT
283.			20	51	F	R	2012	New	SN	NA	NA	NA		CT
284.			35	43	M	U	2012	New	SP	N	N	N		C
285.			27	50	M	R	2012	New	EP	NA	NA	NA		CT
286.			9	19	F	U	2012	New	EP	NA	NA	NA		CT
287.			14	25	M	R	2012	New	SP	NT	NT	N		CT
288.			30	56	F	R	2012	New	EP	NA	NA	NA		CT
289.			1.6	10	F	R	2012	New	EP	NA	NA	NA		CT
290.			25	58	M	R	2012	New	SP	NT	N	N		C
291.			5	14	F	R	2013	New	SN	NA	NA	NA		CT
292.			7	21	M	R	2013	New	SN	NA	NA	NA		CT
293.			40	55	M	U	2013	New	SN	NA	NA	NA		CT
294.			6	14	M	U	2013	New	EP	NA	NA	NA		CT
295.			35	43	M	R	2013	New	SN	NA	NA	NA		CT
296.			13	35	F	U	2013	New	SN	NA	NA	NA		CT
297.			20	44	F	U	2013	New	EP	NA	NA	NA		CT
298.			22	45	M	R	2013	New	SP	N	N	N		C
299.			25	41	M	R	2013	New	EP	NA	NA	NA		CT
300.			11	31	F	R	2013	New	SN	NA	NA	NA		CT
301.			21	43	M	R	2013	New	SN	NA	NA	NA		CT

302.			29	71	M	U	2013	New	EP	NA	NA	NA		CT
303.			14	25	M	R	2013	New	EP	NA	NA	NA		CT
304.			30	48	F	R	2013	New	EP	NA	NA	NA		CT
305.			25	32	F	U	2013	New	EP	NA	NA	NA		CT
306.			30	50	F	R	2013	New	SN	NA	NA	NA		CT
307.			25	45	F	U	2013	New	EP	NA	NA	NA		CT
308.			25	58	M	U	2013	New	SN	NA	NA	NA		CT
309.			28	51	M	U	2013	New	SP	N	N	N		C
310.			26	45	F	U	2013	New	EP	NA	NA	NA		CT
311.			31	38	F	R	2013	New	EP	NA	NA	NA		CT
312.			30	46	F	U	2013	New	EP	NA	NA	NA		CT
313.			7	17	F	U	2013	New	EP	NA	NA	NA		CT
314.			26	34	F	R	2013	New	SP	N	N	N		C
315.			16	46	M	U	2013	New	SN	NA	NA	NA		CT
316.			15	31	F	R	2013	New	EP	NA	NA	NA		CT
317.			38	57	M	R	2013	New	SN	NA	NA	NA		CT
318.			18	50	M	R	2013	New	EP	NA	NA	NA		CT
319.			38	55	M	U	2013	New	SN	NA	NA	NA		CT
320.			17	51	F	U	2013	New	EP	NA	NA	NA		CT
321.			35	30	F	R	2013	RT	SP	N	N	N		Di
322.			26	68	M	U	2013	New	EP	NA	NA	NA		CT
323.			4	10	M	U	2013	New	EP	NA	NA	NA		CT
324.			38	55	M	R	2013	New	SN	NA	NA	NA		CT
325.			22	49	M	U	2013	New	EP	NA	NA	NA		CT
326.			25	38	M	U	2013	New	SP	N	N	N		C
327.			60	39	M	U	2013	New	SN	NA	NA	NA		CT
328.			22	42	M	U	2013	New	SN	NA	NA	NA		CT
329.			21	53	M	U	2013	New	SP	N	N	N		C
330.			3	9	F	R	2013	New	SN	NA	NA	NA		CT
331.			28	50	M	R	2013	New	SN	NA	NA	NA		CT
332.			22	41	F	U	2013	New	EP	NA	NA	NA		CT

333.			18	57	M	U	2013	New	EP	NA	NA	NA		CT
334.			22	8	F	U	2013	New	SN	NA	NA	NA		CT
335.			35	55	M	U	2013	New	SP	N	N	N		C
336.			15	40	F	U	2013	New	EP	NA	NA	NA		CT
337.			35	53	M	U	2013	New	EP	NA	NA	NA		CT
338.			45	32	F	U	2013	New	SP	N	N	N		C
339.			50	58	M	R	2013	Uk	EP	NA	NA	NA		CT
340.			1	10	M	U	2013	New	SN	NA	NA	NA		CT
341.			48	64	M	R	2013	New	EP	NA	NA	NA		CT
342.			65	63	M	U	2013	New	SN	NA	NA	NA		CT
343.			30	58	F	R	2013	New	EP	NA	NA	NA		CT
344.			21	49	M	R	2013	New	SN	NA	NA	NA		CT
345.			25	55	M	R	2013	New	SN	NA	NA	NA		CT
346.			55	64	M	U	2013	New	SN	NA	NA	NA		CT
347.			60	52	M	U	2013	New	EP	NA	NA	NA		CT
348.			59	38	M	R	2013	RT	SP	N	N	N		C
349.			35	48	M	U	2013	New	EP	NA	NA	NA		CT
350.			27	36	M	U	2013	New	SP	N	N	N		C
351.			27	33	F	R	2013	New	SN	NA	NA	NA		CT
352.			20	46	M	R	2013	New	EP	NA	NA	NA		CT
353.			45	44	F	U	2013	New	SP	N	N	N		C
354.			20	50	M	U	2013	New	EP	NA	NA	NA		CT
355.			30	58	M	U	2013	New	SP	N	N	N		C
356.			24	42	F	R	2013	New	EP	NA	NA	NA		CT
357.			28	38	F	U	2013	New	SN	NA	NA	NA		CT
358.			28	46	M	U	2013	New	SP	N	N	N		C
359.			21	40	F	U	2013	New	SN	NA	NA	NA		CT
360.			50	40	F	U	2013	New	SN	NA	NA	NA		CT
361.			30	45	M	U	2013	New	SP	N	N	N		C
362.			25	55	M	U	2013	New	SN	NA	NA	NA		CT
363.			30	32	F	U	2013	New	SN	NA	NA	NA		CT

364.			20	64	M	R	2013	New	EP	NA	NA	NA		CT
365.			45	50	M	U	2013	New	EP	NA	NA	NA		CT
366.			60	45	M	U	2013	New	EP	NA	NA	NA		CT
367.			25	49	M	R	2013	New	EP	NA	NA	NA		CT
368.			39	80	F	R	2014	New	EP	NA	NA	NA		CT
369.			24	43	F	U	2013	New	EP	NA	NA	NA		CT
370.			35	60	M	U	2013	New	EP	NA	NA	NA		CT
371.			45	30	F	U	2013	New	SN	NA	NA	NA		CT
372.			28	51	F	U	2013	New	EP	NA	NA	NA		CT
373.			67	64	M	U	2013	New	EP	NA	NA	NA		CT
374.			25	38	M	R	2013	New	SN	NA	NA	NA		CT
375.			28	45	F	U	2013	New	SP	N	N	N		C
376.			41	56	M	U	2014	New	EP	NA	NA	NA		CT
377.			25	48	F	R	2014	New	SP	N	NT	NT		CT
378.			18	46	M	U	2014	New	EP	NA	NA	NA		CT
379.			20	65	M	R	2014	New	SN	NA	NA	NA		CT
380.			35	46	F	R	2014	New	EP	NA	NA	NA		CT
381.			40	59	M	R	2014	New	EP	NA	NA	NA		CT
382.			60	55	M	R	2014	New	SN	NA	NA	NA		CT
383.			58	40	F	R	2014	New	EP	NA	NA	NA		CT
384.			65	50	M	U	2014	New	SP	N	N	N		C
385.			25	42	F	R	2014	New	EP	NA	NA	NA		CT
386.			27	58	M	R	2014	New	SP	NT	NT	NT		CT
387.			9	20	M	R	2014	New	EP	NA	NA	NA		CT
388.			35	50	F	U	2014	New	SN	NA	NA	NA		CT
389.			35	55	F	R	2014	New	EP	NA	NA	NA		CT
390.			6	17	M	U	2014	New	SN	NA	NA	NA		CT
391.			30	57	M	R	2014	New	SN	NA	NA	NA		CT
392.			5	16	M	U	2014	New	EP	NA	NA	NA		CT
393.			23	57	F	R	2014	Uk	SP	P	P	N		C
394.			50	56	M	R	2014	New	SP	P	N	N		C

395.			26	43	F	R	2014	New	EP	NA	NA	NA		CT
396.			45	56	M	U	2014	New	EP	NA	NA	NA		CT
397.			23	59	F	U	2014	New	SP	N	NT	NT		CT
398.			35	61	M	R	2014	New	EP	NA	NA	NA		CT
399.			38	57	M	U	2014	New	SN	NA	NA	NA		CT
400.			28	32	M	R	2014	New	SP	N	N	N		C
401.			40	60	M	R	2014	New	SN	NA	NA	NA		CT
402.			30	60	M	U	2014	New	EP	NA	NA	NA		CT
403.			0.3	6	F	U	2014	New	EP	NA	NA	NA		CT
404.			46	45	F	R	2014	New	EP	NA	NA	NA		CT
405.			15	47	M	U	2014	New	EP	NA	NA	NA		CT
406.			20	55	M	U	2014	New	EP	NA	NA	NA		CT
407.			61	66	M	R	2014	New	SN	NA	NA	NA		CT
408.			23	42	M	R	2014	New	SN	NA	NA	NA		CT
409.			40	60	M	U	2014	New	SN	NA	NA	NA		CT
410.			15	42	M	U	2014	New	EP	NA	NA	NA		CT
411.			34	64	M	U	2014	New	SP	N	N	N		C
412.			45	56	F	U	2014	New	EP	NA	NA	NA		CT
413.			30	54	M	U	2014	New	SN	NA	NA	NA		CT
414.			70	55	M	U	2014	New	EP	NA	NA	NA		CT
415.			1	10	F	U	2014	New	EP	NA	NA	NA		CT
416.			27	43	F	R	2014	New	EP	NA	NA	NA		CT
417.			15	43	F	U	2014	New	SP	N	N	N		C
418.			30	44	M	U	2014	New	EP	NA	NA	NA		CT
419.			5	14	M	R	2014	New	SN	NA	NA	NA		CT
420.			28	35	M	R	2014	New	SN	NA	NA	NA		CT
421.			27	58	M	R	2014	New	SP	N	N	N		C
422.			36	47	M	U	2014	New	SN	NA	NA	NA		D
423.			50	30	F	U	2014	New	SN	NA	NA	NA		CT
424.			42	45	M	U	2014	New	SN	NA	NA	NA		CT
425.			20	54	F	R	2014	New	EP	NA	NA	NA		CT

426.			12	28	F	U	2014	New	SN	NA	NA	NA		CT
427.			62	40	M	R	2014	New	SN	NA	NA	NA		CT
428.			55	58	M	R	2014	New	SN	NA	NA	NA		CT
429.			12	28	F	U	2014	New	SP	N	N	N		C
430.			50	37	M	R	2014	New	SN	NA	NA	NA		CT
431.			46	45	M	U	2014	New	SN	NA	NA	NA		CT
432.			22	59	F	U	2014	New	SP	N	N	N		C
433.			39	60	M	R	2014	New	SN	NA	NA	NA		CT
434.			30	39	M	R	2014	New	SN	NA	NA	NA		CT
435.			15	42	M	U	2014	New	EP	NA	NA	NA		CT
436.			32	55	M	U	2014	New	SP	N	N	N		C
437.			22	55	M	R	2014	New	EP	NA	NA	NA		CT
438.			18	50	M	U	2014	New	EP	NA	NA	NA		CT
439.			37	50	F	U	2014	New	SN	NA	NA	NA		CT
440.			48	58	M	R	2014	New	EP	NA	NA	NA		CT
441.			8	18	F	R	2014	New	EP	NA	NA	NA		CT
442.			19	46	F	U	2014	New	SP	N	N	N		C
443.			45	62	M	R	2014	New	EP	NA	NA	NA		CT
444.			26	58	F	U	2014	New	SN	NA	NA	NA		CT
445.			25	40	M	U	2014	New	SN	NA	NA	NA		CT
446.			70	54	M	R	2014	New	EP	NA	NA	NA		CT
447.			28	46	F	U	2015	New	SN	NA	NA	NA		CT
448.			20	45	F	R	2015	New	EP	NA	NA	NA		CT
449.			42	61	M	U	2015	New	SN	NA	NA	NA		CT
450.			50	58	F	U	2015	New	SN	NA	NA	NA		CT
451.			24	58	F	R	2015	New	EP	NA	NA	NA		CT
452.			14	36	F	U	2015	New	EP	NA	NA	NA		CT
453.			24	50	M	U	2013	New	SN	NA	NA	NA		CT
454.			27	48	M	U	2015	New	EP	NA	NA	NA		CT
455.			48	55	M	U	2015	New	SN	NA	NA	NA		CT
456.			25	56	M	U	2015	New	SP	N	NT	N		C

457.			36	53	M	U	2015	New	EP	NA	NA	NA		CT
458.			50	42	M	R	2015	New	EP	NA	NA	NA		Di
459.			8	17	M	U	2015	New	EP	NA	NA	NA		CT
460.			50	44	F	U	2015	New	EP	NA	NA	NA		CT
461.			60	55	M	U	2015	New	EP	NA	NA	NA		CT
462.			36	65	M	U	2015	New	EP	NA	NA	NA		CT
463.			45	32	F	R	2015	New	EP	NA	NA	NA		CT
464.			10	8	M	U	2015	New	SN	NA	NA	NA		CT
465.			32	38	F	U	2015	New	EP	NA	NA	NA		CT
466.			28	42	M	U	2015	New	SP	N	N	N		C
467.			26	42	F	R	2015	New	EP	NA	NA	NA		CT
468.			25	41	M	U	2015	New	SP	N	N	N		C
469.			37	55	M	U	2015	New	SN	NA	NA	NA		CT
470.			32	55	F	R	2015	New	EP	NA	NA	NA		CT
471.			28	48	M	R	2015	New	SN	NA	NA	NA		CT
472.			18	44	M	R	2015	New	EP	NA	NA	NA		CT
473.			11	29	M	U	2015	New	EP	NA	NA	NA		CT
474.			28	45	M	R	2015	New	EP	NA	NA	NA		CT
475.			20	50	M	R	2015	New	EP	NA	NA	NA		Di
476.			18	47	F	R	2015	New	EP	NA	NA	NA		CT
477.			28	66	M	R	2015	New	EP	NA	NA	NA		Di
478.			26	45	F	R	2015	New	EP	NA	NA	NA		CT
479.			50	40	F	R	2015	New	SP	N	NT	N		C
480.			55	48	M	R	2015	New	EP	NA	NA	NA		CT
481.			19	53	M	U	2015	New	EP	NA	NA	NA		CT
482.			45	37	M	R	2015	New	SP	NT	NT	NT		Di
483.			13	32	F	R	2015	New	EP	NA	NA	NA		CT
484.			34	59	M	U	2015	New	SP	N	N	N		C
485.			6	10	F	R	2015	New	EP	NA	NA	NA		CT
486.			26	43	M	U	2015	New	EP	NA	NA	NA		CT
487.			35	48	F	R	2015	New	EP	NA	NA	NA		Di

488.			45	45	M	R	2015	New	SN	NA	NA	NA		CT
489.			32	45	F	U	2015	New	EP	NA	NA	NA		CT
490.			25	42	M	R	2015	New	EP	NA	NA	NA		CT
491.			20	56	F	U	2015	New	Uk	NA	NA	NA		CT
492.			16	35	F	U	2015	New	EP	NA	NA	NA		CT
493.			28	60	M	U	2016	New	SN	NA	NA	NA		CT
494.			26	48	M	R	2016	New	EP	NA	NA	NA		CT
495.			10	25	M	R	2016	New	SN	NA	NA	NA		Di
496.			50	59	M	U	2016	New	SN	NA	NA	NA		CT
497.			11	31	F	R	2016	New	EP	NA	NA	NA		CT
498.			28	48	M	U	2016	New	SN	NA	NA	NA		CT
499.			20	41	F	R	2016	New	EP	NA	NA	NA		CT
500.			31	60	M	U	2016	New	SN	NA	NA	NA		CT
501.			12	21	M	R	2016	New	SN	NA	NA	NA		Di
502.			28	35	F	U	2016	New	SP	P	N	N		C
503.			18	51	F	U	2016	New	EP	NA	NA	NA		CT
504.			30	46	M	R	2016	New	SP	NT	NT	NT		Di
505.			13	34	F	U	2016	New	SN	NA	NA	NA		CT
506.			25	43	F	R	2016	New	EP	NA	NA	NA		CT
507.			30	69	M	U	2016	New	EP	NA	NA	NA		Di
508.			37	45	M	R	2016	New	SP	N	N	N		C
509.			74	47	M	U	2016	New	SN	NA	NA	NA		CT
510.			18	44	M	R	2016	New	EP	NA	NA	NA		CT
511.			65	55	F	U	2016	New	SN	NA	NA	NA		CT
512.			30	8	F	R	2016	New	EP	NA	NA	NA		CT
513.			45	45	M	U	2016	New	SP	N	N	N		C
514.			50	46	F	R	2016	New	EP	NA	NA	NA		CT
515.			25	35	F	R	2016	New	SP	N	N	N		C
516.			18	45	M	U	2016	New	SP	N	N	N		C
517.			28	8	F	U	2016	New	EP	NA	NA	NA		CT
518.			40	42	F	U	2016	New	EP	NA	NA	NA		CT

519.			23	40	M	U	2016	New	EP	NA	NA	NA		CT
520.			45	70	F	U	2016	New	EP	NA	NA	NA		Di
521.			50	45	M	R	2016	New	EP	NA	NA	NA		CT
522.			50	65	M	R	2016	New	EP	NA	NA	NA		CT
523.			32	34	F	R	2016	New	EP	NA	NA	NA		CT
524.			40	50	F	R	2016	New	SN	NA	NA	NA		CT
525.			50	38	M	R	2016	New	SN	NA	NA	NA		CT
526.			15	29	F	R	2016	New	EP	NA	NA	NA		Di
527.			21	51	M	R	2016	New	SN	NA	NA	NA		CT
528.			50	52	M	R	2016	New	SP	P	N	N		C
529.			20	45	F	U	2016	New	EP	NA	NA	NA		CT
530.			31	50	M	R	2016	New	SN	NA	NA	NA		CT
531.			30	55	F	U	2016	New	SP	N	N	NT		C
532.			3	16	F	U	2016	New	EP	NA	NA	NA		CT
533.			25	50	M	U	2016	New	SP	N	N	N		C
534.			45	56	F	R	2016	New	EP	NA	NA	NA		CT
535.			50	48	M	U	2016	New	SN	NA	NA	NA		CT
536.			52	55	M	U	2016	New	SP	N	N	N		C
537.			20	51	M	R	2016	New	EP	NA	NA	NA		CT
538.			50	52	M	U	2016	New	SP	N	N	N		C
539.			52	58	M	R	2016	New	EP	NA	NA	NA		CT
540.			24	54	M	U	2016	New	EP	NA	NA	NA		CT
541.			17	50	M	R	2016	New	EP	NA	NA	NA		Di
542.			56	49	F	U	2016	New	SN	NA	NA	NA		CT
543.			19	50	M	R	2016	New	EP	NA	NA	NA		CT
544.			17	48	M	U	2016	New	EP	NA	NA	NA		CT
545.			30	45	M	U	2016	New	EP	NA	NA	NA		CT
546.			30	54	M	R	2016	New	EP	NA	NA	NA		CT
547.			55	34	M	U	2016	New	SP	NT	NT	NT		C
548.			0.8	7	F	U	2016	New	SN	NA	NA	NA		CT
549.			55	35	F	U	2016	RT	SN	NA	NA	NA		CT

550.			19	48	M	U	2016	New	SN	NA	NA	NA		NE
551.			29	50	F	U	2016	New	EP	NA	NA	NA		CT
552.			15	53	F	U	2016	New	EP	NA	NA	NA		CT
553.			30	50	F	R	2016	New	EP	NA	NA	NA		CT
554.			25	45	F	U	2016	New	SP	N	N	N		C
555.			45	70	M	U	2016	New	SN	NA	NA	NA		CT
556.			20	36	M	U	2016	New	SP	N	NT	NT		Di
557.			18	49	M	U	2016	New	SN	NA	NA	NA		CT
558.			25	42	F	R	2016	New	EP	NA	NA	NA		CT
559.			14	31	F	R	2016	New	SN	NA	NA	NA		CT
560.			6	16	M	R	2016	New	SN	NA	NA	NA		CT
561.			14	35	M	U	2016	New	EP	NA	NA	NA		CT
562.			15	53	F	U	2016	New	SN	NA	NA	NA		CT
563.			42	36	F	U	2016	RT	SN	NA	NA	NA		Di
564.			40	34	F	U	2016	New	SP	N	N	N		C
565.			45	75	F	R	2016	New	EP	NA	NA	NA		CT
566.			36	58	M	R	2016	New	EP	NA	NA	NA		CT
567.			18	41	F	R	2016	New	SN	NA	NA	NA		CT
568.			25	54	M	R	2016	New	SP	N	N	N		C
569.			40	53	F	U	2016	New	EP	NA	NA	NA		NE
570.			31	56	M	R	2016	RT	SP	P	P	P		TR
571.			35	38	M	R	2016	New	SP	N	N	N		C
572.			56	42	F	R	2016	New	EP	NA	NA	NA		CT
573.			30	32	F	R	2016	New	EP	NA	NA	NA		CT
574.			55	57	M	U	2016	New	SN	NA	NA	NA		CT
575.			35	32	F	R	2016	New	EP	NA	NA	NA		CT
576.			67	57	M	8	2016	New	SN	NA	NA	NA		CT
577.			16	44	F	U	2016	New	EP	NA	NA	NA		Di
578.			37	38	F	R	2016	New	SP	N	N	N		C
579.			5	14	F	U	2016	New	EP	NA	NA	NA		CT
580.			25	57	M	U	2016	New	EP	NA	NA	NA		CT

581.			31	47	F	U	2016	New	SP	N	N	N		C
582.			28	45	M	U	2016	New	SP	N	N	N		C
583.			17	42	F	U	2016	New	SP	N	N	N		C
584.			10	23	F	U	2016	New	SP	N	N	N		NE
585.			13	22	M	R	2016	New	EP	NA	NA	NA		CT
586.			28	59	M	R	2016	New	SP	N	NT	N		C
587.			25	60	F	U	2016	New	SP	N	N	N		C
588.			33	33	F	R	2016	New	SP	N	N	N		CT
589.			34	41	M	U	2016	New	EP	NA	NA	NA		CT
590.			48	45	F	R	2016	New	SP	N	NT	N		C
591.			10	28	M	U	2016	New	SP	N	NT	N		C
592.			22	57	M	R	2016	New	EP	NA	NA	NA		CT
593.			18	50	F	U	2016	New	SP	P	P	P		TR
594.			50	53	M	U	2016	New	SN	NA	NA	NA		CT
595.			55	59	M	U	2016	New	SP	N	N	N		C
596.			17	47	M	U	2016	New	SN	NA	NA	NA		CT
597.			8	22	M	R	2016	New	EP	NA	NA	NA		NE
598.			22	54	M	U	2016	New	SN	NA	NA	NA		CT
599.			15	41	M	U	2016	New	EP	NA	NA	NA		CT
600.			50	48	M	R	2016	New	SN	NA	NA	NA		Di
601.			13	28	M	U	2016	New	SP	N	N	N		C
602.			43	52	M	U	2016	New	SP	N	N	N		C
603.			25	50	F	R	2016	New	EP	NA	NA	NA		CT
604.			26	52	M	R	2016	New	SP	N	NT	N		C
605.			30	54	M	R	2016	New	SN	NA	NA	NA		CT
606.			18	43	M	U	2016	New	SN	NA	NA	NA		CT
607.			24	58	M	U	2016	New	SP	N	N	N		C
608.			33	33	M	U	2016	New	SP	NT	NT	NT		Di
609.			21	45	M	U	2016	New	SP	N	NT	NT		CT
610.			8	22	M	R	2016	New	SN	NA	NA	NA		CT
611.			20	50	M	U	2016	New	SP	N	NT	N		C

612.			80	37	M	U	2016	New	SP	N	NT	N		C
613.			60	44	F	U	2016	New	SP	N	NT	N		C
614.			32	47	M	U	2016	New	SP	P	P	N		C
615.			7	19	F	R	2016	New	SP	N	NT	N		C
616.			35	45	F	U	2016	New	SP	N	N	N		C
617.			13	34	M	U	2016	New	EP	NA	NA	NA		CT
618.			16	48	M	U	2016	New	SP	N	N	N		C
619.			13	60	M	R	2016	New	EP	NA	NA	NA		CT
620.			70	40	F	R	2016	New	EP	NA	NA	NA		CT
621.			34	53	M	U	2016	New	SN	NA	NA	NA		CT
622.			38	43	M	U	2016	New	SN	NA	NA	NA		CT
623.			43	48	M	R	2016	New	EP	NA	NA	NA		CT
624.			40	52	M	R	2016	New	SN	NA	NA	NA		CT
625.			30	55	M	U	2016	New	EP	NA	NA	NA		CT
626.			34	51	M	U	2016	New	SN	NA	NA	NA		CT
627.			45	54	F	R	2016	New	SN	NA	NA	NA		CT
628.			48	55	M	U	2016	New	SN	NA	NA	NA		CT
629.			60	47	M	R	2016	New	EP	NA	NA	NA		CT
630.			17	42	M	U	2016	New	EP	NA	NA	NA		CT
631.			40	45	F	U	2016	New	SP	N	N	N		C
632.			16	46	M	R	2016	New	SP	N	N	N		C
633.			35	41	F	U	2012	New	EP	NA	NA	NA		CT
634.			4.5	14	M	R	2012	New	EP	NA	NA	NA		CT
635.			13	30	M	R	2012	New	SN	NA	NA	NA		CT
636.			15	38.5	F	R	2012	New	EP	NA	NA	NA		CT
637.			40	52	M	R	2012	New	SN	NA	NA	NA		CT
638.			25	55	M	U	2012	New	SP	N	N	N		C
639.			15	39	F	R	2012	New	SP	N	N	N		C
640.			12	20	F	R	2012	New	EP	NA	NA	NA		CT
641.			57	56	M	U	2012	New	SN	NA	NA	NA		CT
642.			22	63.3	M	R	2012	New	SN	NA	NA	NA		CT

643.			23	46.5	F	R	2012	New	SP	NT	NT	NT		CT
644.			19	48	M	U	2012	New	EP	NA	NA	NA		CT
645.			27	51	M	R	2012	New	EP	NA	NA	NA		CT
646.			25	49	M	R	2012	New	EP	NA	NA	NA		CT
647.			50	49	M	R	2012	New	SN	NA	NA	NA		NE
648.			50	50	F	R	2012	New	SN	NA	NA	NA		CT
649.			58	49	F	U	2012	New	EP	NA	NA	NA		CT
650.			60	55	M	R	2012	New	SN	NA	NA	NA		NE
651.			45	64	M	U	2012	New	SN	NA	NA	NA		CT
652.			32	37	F	R	2012	New	SP	P	N	N		C
653.			32	67	M	U	2012	New	EP	NA	NA	NA		CT
654.			5	15.5	F	R	2012	New	SN	NA	NA	NA		CT
655.			18	45	F	R	2012	New	EP	NA	NA	NA		CT
656.			13	40	M	R	2012	New	SN	NA	NA	NA		CT
657.			28	50	F	U	2012	New	SN	NA	NA	NA		CT
658.			24	111	F	U	2012	New	SN	NA	NA	NA		CT
659.			30	50	F	R	2012	New	EP	NA	NA	NA		CT
660.			30	50	M	R	2012	New	SP	N	NT	NT		CT
661.			21	52	M	U	2012	New	SP	N	NT	NT		CT
662.			25	51.5	M	R	2012	New	EP	NA	NA	NA		CT
663.			27	45	F	R	2012	New	SP	N	N	N		C
664.			25	54	F	U	2012	New	EP	NA	NA	NA		CT
665.			22	53	M	R	2012	New	SN	NA	NA	NA		Di
666.			49	80	F	R	2012	New	EP	NA	NA	NA		CT
667.			45	42	F	R	2012	New	SN	NA	NA	NA		CT
668.			15	29	F	R	2012	New	SP	N	N	N		C
669.			34	66	M	R	2012	New	SP	N	N	N		C
670.			28	45	F	R	2012	New	EP	NA	NA	NA		CT
671.			45	45	M	R	2012	New	SN	NA	NA	NA		CT
672.			16	32	F	R	2012	New	EP	NA	NA	NA		CT
673.			40	41	F	U	2012	New	EP	NA	NA	NA		CT

674.			12	40	F	R	2012	New	EP	NA	NA	NA		CT
675.			27	50	F	R	2012	New	SN	NA	NA	NA		CT
676.			55	38	M	R	2012	New	SN	NA	NA	NA		D
677.			38	51	F	U	2012	New	SN	NA	NA	NA		CT
678.			50	54	M	U	2012	New	SN	NA	NA	NA		Di
679.			75	38.5	F	U	2012	New	SP	N	N	N		C
680.			60	59	M	R	2012	New	SN	NA	NA	NA		Di
681.			30	48	M	R	2012	New	EP	NA	NA	NA		CT
682.			63	41	F	U	2012	New	SN	NA	NA	NA		CT
683.			40	53.5	M	U	2012	New	SN	NA	NA	NA		CT
684.			37	55	M	R	2012	New	SN	NA	NA	NA		CT
685.			28	40	F	R	2012	New	EP	NA	NA	NA		CT
686.			42	52	M	R	2012	New	SN	NA	NA	NA		CT
687.			45	57	M	R	2012	New	SN	NA	NA	NA		CT
688.			20	52	M	R	2012	New	EP	NA	NA	NA		CT
689.			45	54	M	R	2012	New	EP	NA	NA	NA		CT
690.			50	40	F	R	2012	New	EP	NA	NA	NA		CT
691.			50	56	F	U	2012	New	EP	NA	NA	NA		CT
692.			65	50	M	R	2012	New	SN	NA	NA	NA		CT
693.			35	57	F	U	2012	New	SP	N	N	N		CT
694.			56	53	M	R	2012	New	EP	NA	NA	NA		Di
695.			35	41	F	U	2012	New	EP	NA	NA	NA		CT
696.			28	40	F	R	2012	New	SP	N	N	N		C
697.			18	50	M	U	2012	New	SP	N	N	N		C
698.			46	46	M	R	2012	New	EP	NA	NA	NA		Di
699.			47	47	M	R	2012	New	EP	NA	NA	NA		CT
700.			23	49	F	U	2012	New	SN	NA	NA	NA		CT
701.			5	15	F	U	2012	New	SN	NA	NA	NA		CT
702.			30	54	F	U	2012	New	EP	NA	NA	NA		CT
703.			50	49	M	U	2012	New	SN	NA	NA	NA		CT
704.			11	29	M	8	2012	New	EP	NA	NA	NA		C

705.			20	53	M	R	2012	New	SP	N	N	N		C
706.			10	20	F	U	2012	New	EP	NA	NA	NA		CT
707.			25	55	F	U	2012	New	EP	NA	NA	NA		CT
708.			12	33	F	U	2012	New	EP	NA	NA	NA		CT
709.			70	49	M	R	2012	New	SN	NA	NA	NA		CT
710.			30	55	M	R	2013	New	SP	N	N	N		C
711.			18	49	M	U	2013	New	SP	N	N	N		C
712.			82	39	M	U	2013	New	EP	NA	NA	NA		CT
713.			31	41	F	R	2013	New	SN	NA	NA	NA		CT
714.			31	52	M	U	2013	RT	EP	NA	NA	NA		CT
715.			46	40	F	U	2013	New	EP	NA	NA	NA		CT
716.			24	55	F	R	2013	New	EP	NA	NA	NA		CT
717.			50	51	F	R	2013	New	EP	NA	NA	NA		CT
718.			15	29	F	R	2013	RT	SP	N	N	N		C
719.			36	33	F	R	2013	New	EP	NA	NA	NA		CT
720.			13	30	F	R	2013	New	EP	NA	NA	NA		CT
721.			17	41	F	R	2013	New	EP	NA	NA	NA		CT
722.			25	41	F	R	2013	New	EP	NA	NA	NA		CT
723.			35	45	F	R	2013	New	EP	NA	NA	NA		CT
724.			2	7	F	R	2013	New	EP	NA	NA	NA		CT
725.			12	29	M	U	2013	New	EP	NA	NA	NA		CT
726.			65	46	M	U	2013	New	EP	NA	NA	NA		CT
727.			19	42	F	R	2013	New	SN	NA	NA	NA		CT
728.			45	37	F	R	2013	New	SN	NA	NA	NA		CT
729.			45	35	F	R	2013	New	EP	NA	NA	NA		CT
730.			56	50	M	R	2013	New	SN	NA	NA	NA		CT
731.			23	66.5	F	U	2013	New	EP	NA	NA	NA		CT
732.			30	40.5	M	R	2013	New	EP	NA	NA	NA		CT
733.			10	34.5	F	R	2013	New	EP	NA	NA	NA		CT
734.			8	27	F	R	2013	New	EP	NA	NA	NA		CT
735.			20	41	M	R	2013	New	SP	NT	NT	NT		Di

736.			30	41	M	U	2013	New	SN	NA	NA	NA		CT
737.			31	43	M	R	2013	New	SN	NA	NA	NA		CT
738.			32	49	M	U	2013	New	EP	NA	NA	NA		CT
739.			7	20	M	U	2013	New	EP	NA	NA	NA		CT
740.			85	44	M	R	2013	New	SN	NA	NA	NA		CT
741.			50	55	M	R	2013	New	SN	NA	NA	NA		CT
742.			4	12.5	M	U	2013	New	EP	NA	NA	NA		D
743.			25	64	M	U	2013	New	SN	NA	NA	NA		CT
744.			35	61	F	U	2013	RT	SN	NA	NA	NA		CT
745.			18	64	M	R	2013	New	SN	NA	NA	NA		CT
746.			60	45	F	R	2013	New	SN	NA	NA	NA		CT
747.			23	56	F	U	2013	New	SN	NA	NA	NA		CT
748.			35	56	M	R	2013	New	EP	NA	NA	NA		CT
749.			25	48	F	R	2013	New	EP	NA	NA	NA		CT
750.			34	50	M	R	2013	New	EP	NA	NA	NA		CT
751.			15	49	F	U	2013	New	SN	NA	NA	NA		CT
752.			30	58	M	U	2013	New	SP	N	N	N		C
753.			80	38	F	U	2013	RT	SN	NA	NA	NA		CT
754.			5	15.5	M	R	2013	New	EP	NA	NA	NA		CT
755.			35	69	M	R	2013	New	SN	NA	NA	NA		CT
756.			48	50	M	U	2013	New	SN	NA	NA	NA		CT
757.			35	56	F	U	2013	New	EP	NA	NA	NA		CT
758.			25	65	M	R	2013	New	EP	NA	NA	NA		CT
759.			17	40	F	R	2013	New	SP	N	N	N		C
760.			4	12	M	R	2013	New	EP	NA	NA	NA		CT
761.			35	56	M	U	2013	RT	SN	NA	NA	NA		CT
762.			6	38	F	R	2013	New	SN	NA	NA	NA		Di
763.			12	22	F	U	2013	New	EP	NA	NA	NA		CT
764.			45	38	M	R	2013	New	EP	NA	NA	NA		CT
765.			21	51	M	R	2013	Uk	EP	NA	NA	NA		CT
766.			16	51	M	R	2013	New	SP	N	NT	N		C

767.			20	56	M	U	2013	New	SP	N	NT	N		C
768.			41	51	F	U	2013	New	SN	NA	NA	NA		CT
769.			6	45	F	R	2013	New	SN	NA	NA	NA		CT
770.			60	45	M	R	2013	New	SN	NA	NA	NA		CT
771.			27	42	F	U	2013	New	SP	N	N	NT		C
772.			45	44	M	U	2013	RT	SN	NA	NA	NA		CT
773.			60	43	F	R	2013	New	EP	NA	NA	NA		CT
774.			28	39	F	R	2013	New	EP	NA	NA	NA		CT
775.			45	57	F	U	2013	New	EP	NA	NA	NA		CT
776.			45	39	M	R	2013	New	EP	NA	NA	NA		CT
777.			40	44	M	R	2013	New	SN	NA	NA	NA		Di
778.			55	58	F	U	2013	New	EP	NA	NA	NA		CT
779.			28	46	M	R	2013	New	EP	NA	NA	NA		Di
780.			22	48	F	R	2013	New	EP	NA	NA	NA		CT
781.			18	48	M	R	2013	New	EP	NA	NA	NA		CT
782.			10	27	F	U	2013	New	EP	NA	NA	NA		CT
783.			18	56	M	U	2013	New	EP	NA	NA	NA		CT
784.			8	22	M	U	2013	New	EP	NA	NA	NA		CT
785.			22	50	M	U	2013	New	EP	NA	NA	NA		CT
786.			18	45	F	R	2014	New	EP	NA	NA	NA		Di
787.			40	52	M	R	2014	New	SN	NA	NA	NA		CT
788.			60	48.5	F	U	2014	New	EP	NA	NA	NA		NE
789.			40	63	M	U	2014	New	SN	NA	NA	NA		CT
790.			20	54	M	U	2014	New	EP	NA	NA	NA		CT
791.			75	38	F	U	2014	New	SN	NA	NA	NA		CT
792.			17	49	M	U	2014	New	SP	N	N	NT		C
793.			53	35	M	U	2014	New	SP	N	N	NT		C
794.			18	41	F	U	2014	New	SP	NT	NT	NT		Di
795.			30	41	F	R	2014	New	SN	NA	NA	NA		CT
796.			25	43	M	R	2014	New	EP	NA	NA	NA		NE
797.			18	50	F	R	2014	New	EP	NA	NA	NA		NE

798.			37	45	F	R	2014	New	SP	N	NT	NT		CT
799.			46	50	M	R	2014	New	SN	NA	NA	NA		CT
800.			40	43	M	R	2014	New	EP	NA	NA	NA		CT
801.			74	52	M	R	2014	New	EP	NA	NA	NA		D
802.			53	61	M	R	2014	New	SN	NA	NA	NA		CT
803.			32	75	F	U	2014	New	SP	N	N	N		C
804.			25	41	F	R	2014	New	EP	NA	NA	NA		CT
805.			80	52	M	R	2014	New	EP	NA	NA	NA		CT
806.			27	49	M	U	2014	New	SP	N	N	NT		C
807.			45	51	M	R	2014	New	SN	NA	NA	NA		C
808.			20	68	M	U	2014	New	EP	NA	NA	NA		CT
809.			32	42.5	F	R	2014	New	SP	N	N	NT		C
810.			44	47	F	U	2014	RT	SN	NA	NA	NA		CT
811.			30	45	M	R	2014	New	SP	N	N	N		C
812.			18	3	F	R	2014	New	SN	NA	NA	NA		CT
813.			40	68	M	R	2014	New	EP	NA	NA	NA		CT
814.			60	48	M	R	2014	New	SN	NA	NA	NA		CT
815.			51	47	M	R	2014	New	SN	NA	NA	NA		CT
816.			23	50	M	R	2014	New	EP	NA	NA	NA		CT
817.			65	38	F	R	2014	New	EP	NA	NA	NA		Di
818.			20	45	F	R	2014	New	SN	NA	NA	NA		CT
819.			40	67	F	R	2014	New	SN	NA	NA	NA		CT
820.			34	50	M	R	2014	New	SP	N	N	N		C
821.			59	53	M	U	2014	New	EP	NA	NA	NA		CT
822.			30	81	M	R	2014	New	EP	NA	NA	NA		CT
823.			25	35	F	U	2014	New	SN	NA	NA	NA		CT
824.			50	51	F	R	2014	New	SP	N	N	N		C
825.			50	45	F	U	2014	New	EP	NA	NA	NA		CT
826.			60	52	M	R	2014	New	SP	N	N	N		C
827.			60	44	M	R	2014	New	EP	NA	NA	NA		CT
828.			70	47	M	R	2014	New	EP	NA	NA	NA		Di

829.			45	42	F	R	2014	New	EP	NA	NA	NA		CT
830.			30	43	M	U	2014	New	SN	NA	NA	NA		D
831.			60	59	F	U	2014	New	EP	NA	NA	NA		CT
832.			25	55	M	R	2014	New	EP	NA	NA	NA		CT
833.			15	35	M	R	2014	New	EP	NA	NA	NA		CT
834.			20	59	M	R	2014	New	EP	NA	NA	NA		CT
835.			19	55.4	M	R	2014	New	EP	NA	NA	NA		CT
836.			44	50.5	F	U	2014	RT	EP	NA	NA	NA		CT
837.			30	54.5	M	R	2014	New	EP	NA	NA	NA		CT
838.			73	52	M	U	2014	New	EP	NA	NA	NA		CT
839.			35	49.5	F	R	2014	New	EP	NA	NA	NA		CT
840.			20	40	F	U	2014	New	EP	NA	NA	NA		CT
841.			30	57	M	U	2014	New	SP	N	NT	NT		CT
842.			24	58	M	U	2014	New	EP	NA	NA	NA		CT
843.			25	52	F	R	2014	New	EP	NA	NA	NA		CT
844.			38	38	F	R	2014	New	SN	NA	NA	NA		CT
845.			25	55	M	R	2014	New	EP	NA	NA	NA		CT
846.			22	53	M	R	2014	New	EP	NA	NA	NA		CT
847.			20	46	F	U	2014	New	EP	NA	NA	NA		CT
848.			50	45	M	R	2014	RT	SN	NA	NA	NA		Di
849.			65	42	F	R	2014	New	SN	NA	NA	NA		CT
850.			8	22	M	R	2014	New	SN	NA	NA	NA		CT
851.			22	63	M	R	2014	New	EP	NA	NA	NA		CT
852.			50	44	M	U	2014	RT	SN	NA	NA	NA		CT
853.			42	43	M	R	2014	New	SN	NA	NA	NA		CT
854.			33	62	M	R	2014	New	SN	NA	NA	NA		Di
855.			23	53	M	U	2014	New	EP	NA	NA	NA		CT
856.			50	45	F	R	2014	New	SN	NA	NA	NA		CT
857.			45	40	F	R	2015	New	SN	NA	NA	NA		CT
858.			53	42	M	R	2015	New	SP	N	N	N		C
859.			76	43	M	R	2015	New	SN	NA	NA	NA		CT

860.			26	35	F	R	2015	New	SN	NA	NA	NA		CT
861.			65	38	F	U	2015	New	SN	NA	NA	NA		CT
862.			36	43	M	R	2015	New	SN	NA	NA	NA		CT
863.			15	43	F	R	2015	New	SN	NA	NA	NA		Di
864.			56	34.5	F	U	2015	New	SN	NA	NA	NA		CT
865.			3	7	M	U	2015	New	SN	NA	NA	NA		D
866.			35	54	F	R	2015	New	EP	NA	NA	NA		CT
867.			35	54.5	F	R	2015	New	SN	NA	NA	NA		CT
868.			35	39	F	R	2015	New	SN	NA	NA	NA		CT
869.			35	40	M	R	2015	New	EP	NA	NA	NA		CT
870.			52	45	M	R	2015	New	EP	NA	NA	NA		CT
871.			60	60	M	R	2015	New	EP	NA	NA	NA		CT
872.			42	52	M	R	2015	New	SP	N	N	N		C
873.			1.5	10	M	U	2015	New	EP	NA	NA	NA		CT
874.			18	53	M	R	2015	New	SN	NA	NA	NA		CT
875.			80	49	M	R	2015	New	EP	NA	NA	NA		CT
876.			73	50	M	U	2015	New	SP	N	N	N		C
877.			40	50	F	U	2015	New	EP	NA	NA	NA		Di
878.			5	14	F	R	2015	New	EP	NA	NA	NA		CT
879.			50	45	F	R	2015	New	SN	NA	NA	NA		CT
880.			45	42	F	R	2015	New	EP	NA	NA	NA		CT
881.			37	65	M	U	2015	New	EP	NA	NA	NA		CT
882.			28	69	M	U	2015	New	SP	N	N	N		C
883.			70	46	F	R	2015	New	SN	NA	NA	NA		CT
884.			25	46	F	R	2015	New	EP	NA	NA	NA		CT
885.			15	31	F	R	2015	New	EP	NA	NA	NA		CT
886.			22	58	M	U	2015	New	SP	N	N	N		C
887.			23	46	M	R	2015	New	SN	NA	NA	NA		NE
888.			57	46.5	M	U	2015	New	SP	N	N	N		C
889.			70	45	M	R	2015	New	SN	NA	NA	NA		CT
890.			18	55	F	U	2015	8	SP	N	N	N		C

891.			45	36	F	8	2015	New	SN	NA	NA	NA		CT
892.			31	54	M	U	2015	RT	SP	N	NT	NT		CT
893.			65	47	F	U	2015	New	EP	NA	NA	NA		CT
894.			55	47	M	R	2015	RT	SP	N	N	N		C
895.			18	41.5	F	U	2015	New	SN	NA	NA	NA		CT
896.			39	55	M	R	2015	New	EP	NA	NA	NA		CT
897.			50	55	M	R	2015	New	SN	NA	NA	NA		Di
898.			32	49	F	U	2015	New	EP	NA	NA	NA		CT
899.			55	54	M	R	2015	New	EP	NA	NA	NA		CT
900.			25	46	F	R	2015	New	EP	NA	NA	NA		CT
901.			28	36	F	R	2015	New	SN	NA	NA	NA		CT
902.			27	46	F	U	2015	New	EP	NA	NA	NA		CT
903.			20	40.5	F	R	2015	New	EP	NA	NA	NA		CT
904.			28	46.3	F	R	2015	New	EP	NA	NA	NA		CT
905.			38	71	M	U	2015	New	SP	N	N	N		C
906.			39	50.5	M	U	2015	New	SN	NA	NA	NA		CT
907.			50	54	M	R	2015	New	SN	NA	NA	NA		Di
908.			30	67	M	U	2015	New	SN	NA	NA	NA		CT
909.			35	44	M	U	2015	New	SP	N	N	N		C
910.			52	36.6	F	R	2015	New	EP	NA	NA	NA		Di
911.			60	52	F	R	2015	New	SN	NA	NA	NA		NE
912.			40	49	M	U	2015	New	EP	NA	NA	NA		NE
913.			47	63	M	R	2015	New	SN	NA	NA	NA		CT
914.			40	33.5	F	R	2015	New	SP	N	N	N		C
915.			11	29	F	R	2015	New	EP	NA	NA	NA		CT
916.			17	52.3	M	R	2015	New	SN	NA	NA	NA		CT
917.			45	35	M	R	2015	New	SN	NA	NA	NA		Di
918.			54	44	M	R	2015	New	SN	NA	NA	NA		CT
919.			35	54	M	U	2015	RT	SN	NA	NA	NA		CT
920.			55	47.5	M	R	2015	New	SN	NA	NA	NA		D
921.			20	46.5	M	R	2015	New	EP	NA	NA	NA		CT

922.			46	36	F	R	2015	New	SP	N	N	N		C
923.			65	40	F	R	2015	New	SN	NA	NA	NA		CT
924.			40	49	M	R	2015	New	EP	NA	NA	NA		CT
925.			35	47.8	F	U	2015	New	SN	NA	NA	NA		CT
926.			40	46	F	U	2015	RT	EP	NA	NA	NA		CT
927.			15	41.4	F	R	2015	New	EP	NA	NA	NA		CT
928.			11	25	M	U	2015	New	SN	NA	NA	NA		CT
929.			10	27	M	R	2015	New	EP	NA	NA	NA		CT
930.			27	55.5	M	R	2015	New	SP	N	N	N		C
931.			45	41	F	R	2015	RT	SN	NA	NA	NA		CT
932.			70	38	M	R	2015	New	SP	N	N	NT		TR
933.			55	51	M	U	2015	New	SN	NA	NA	NA		CT
934.			45	52	M	R	2015	RT	SN	NA	NA	NA		D
935.			45	50	F	R	2015	New	SN	NA	NA	NA		CT
936.			35	45	F	R	2015	New	SN	NA	NA	NA		CT
937.			87	35.9	M	U	2015	RT	SN	NA	NA	NA		Di
938.			75	38.5	M	R	2015	New	EP	NA	NA	NA		CT
939.			35	61	M	R	2015	New	EP	NA	NA	NA		CT
940.			13	34	M	R	2016	New	EP	NA	NA	NA		CT
941.			20	55	M	R	2016	New	SN	NA	NA	NA		CT
942.			16	36	F	R	2016	New	SN	NA	NA	NA		D
943.			13	34	M	R	2016	New	EP	NA	NA	NA		CT
944.			24	57	M	U	2016	New	SN	NA	NA	NA		CT
945.			39	60	M	U	2016	New	SN	NA	NA	NA		CT
946.			28	50	M	R	2016	New	SP	N	N	N		C
947.			35	46	M	U	2016	New	EP	NA	NA	NA		CT
948.			27	55	F	R	2016	New	SN	NA	NA	NA		CT
949.			45	58	F	U	2016	New	SN	NA	NA	NA		CT
950.			20	48	M	R	2016	New	EP	NA	NA	NA		CT
951.			13	40	F	R	2016	New	SN	NA	NA	NA		CT
952.			40	40	F	R	2016	New	SN	NA	NA	NA		CT

953.			36	40	F	U	2016	New	EP	NA	NA	NA		CT
954.			12	34	F	R	2016	New	SP	P	N	N		C
955.			45	38	F	R	2016	New	EP	NA	NA	NA		CT
956.			40	44	F	U	2016	New	EP	NA	NA	NA		CT
957.			29	57	M	U	2016	New	EP	NA	NA	NA		CT
958.			51	54	M	R	2016	New	SN	NA	NA	NA		CT
959.			26	51	M	R	2016	New	EP	NA	NA	NA		Di
960.			26	42	F	U	2016	New	SN	NA	NA	NA		CT
961.			18	64	M	R	2016	New	EP	NA	NA	NA		CT
962.			22	46.5	M	R	2016	New	SN	NA	NA	NA		CT
963.			22	64	M	R	2016	New	EP	NA	NA	NA		CT
964.			23	45	F	U	2016	New	SN	NA	NA	NA		CT
965.			50	50	M	U	2016	New	SN	NA	NA	NA		CT
966.			35	48.5	M	R	2016	New	EP	NA	NA	NA		CT
967.			22	45	F	U	2016	New	EP	NA	NA	NA		CT
968.			45	45	F	R	2016	New	SN	NA	NA	NA		CT
969.			48	56	M	U	2016	New	EP	NA	NA	NA		CT
970.			22	50	M	U	2016	New	SN	NA	NA	NA		CT
971.			25	49	M	R	2016	New	EP	NA	NA	NA		CT
972.			6	15	M	R	2016	New	EP	NA	NA	NA		CT
973.			32	50	M	U	2016	New	SN	NA	NA	NA		Di
974.			14	40	M	R	2016	New	SN	NA	NA	NA		CT
975.			42	55.5	M	R	2016	New	SN	NA	NA	NA		CT
976.			18	43.5	M	U	2016	New	SP	P	N	N		C
977.			45	50	M	R	2016	New	EP	NA	NA	NA		CT
978.			9	25	M	R	2016	New	EP	NA	NA	NA		CT
979.			17	40	M	U	2016	RT	SN	NA	NA	NA		CT
980.			30	62.5	M	U	2016	New	SN	NA	NA	NA		CT
981.			18	46.5	M	R	2016	New	SP	N	N	N		C
982.			58	67	M	R	2016	New	SN	NA	NA	NA		CT
983.			4	12	M	R	2016	New	EP	NA	NA	NA		CT

984.			40	40	F	R	2016	New	EP	NA	NA	NA		CT
985.			19	64	M	R	2016	New	EP	NA	NA	NA		CT
986.			22	59.5	M	U	2016	New	SP	N	N	N		C
987.			35	45	F	U	2016	New	SN	NA	NA	NA		CT
988.			20	52	F	U	2016	New	SN	NA	NA	NA		CT
989.			45	39.5	F	R	2016	New	EP	NA	NA	NA		CT
990.			65	45	M	R	2016	New	EP	NA	NA	NA		CT
991.			30	50	F	R	2016	New	EP	NA	NA	NA		CT
992.			54	47	M	R	2016	RT	SN	NA	NA	NA		NE
993.			40	50	M	R	2016	New	EP	NA	NA	NA		CT
994.			20	57.5	F	R	2016	New	EP	NA	NA	NA		CT
995.			23	50	F	U	2016	New	EP	NA	NA	NA		CT

UHC, Uke health center; SSHC, Sibu Sire health center; AGHC, Anger Gute health center; M, male; F, female; U, urban; R, rural; RT, Re-treated; SP, smear-positive; SN, smear-negative; EP, extrapulmonary; P, positive, N, negative; NA, not applicable, NE, not evaluated; NT, not tested; C, cured; CT, completed treatment; D, defaulted; Di, died.
